# Current Awareness on Comparative and Functional Genomics

**DOI:** 10.1002/cfg.56

**Published:** 2001-04


**Comparative and Functional Genomics**
*Comp. Funct. Genom.* 2001; **2:** 115–122
DOI:10.1002/cfg.56

### Current awareness on comparative and functional genomics

### 1 Reviews & symposia

2000. Special issue: Pharmacogenetics and pharmacogenomics - Recent conceptual and technical advances. *Pharmacology* **61:** (3)

2000. Special issue: Proteomics forum '99: Proceedings of a meeting of the German Electrophoresis Society - Munich, Germany, October 25-27, 1999. *Electrophoresis* **21:** (13)

Alexandrova EM, Zaraisky AG. 2000. Russian Acad Sci, Shemyakin & Ovchinnikov Inst Bioorgan Chem, RU-117871 Moscow, Russia. Molecular mechanisms of early neurogenesis in vertebrates. *Mol Biol-Engl Tr* **34:** (4) 496.

Andersen JS, Mann M*. 2000. *Univ Sthn Denmark, Protein Interact Lab, DK-5230 Odense M, Denmark. Functional genomics by mass spectrometry (Minireview). *FEBS Lett* **480:** (1) 25.

Anderson NL, Matheson AD, Steiner S. 2000. Large Scale Proteomics Corp, 9620 Med Ctr Dr, Rockville, Md 20850, USA. Proteomics: Application in basic and applied biology. *Curr Opin Biotechnol* **11:** (4) 408.

Baldi P, Brunak S, Chauvin Y, Andersen CAF, Nielsen H. 2000. Univ Calif, Dept Informat & Comp Sci, Irvine, Ca 92697, USA. Assessing the accuracy of prediction algorithms for classification: An overview. *Bioinformatics* **16:** (5) 412.

Bancroft I. 2000. John Innes Ctr, Norwich Res Park, Colney, Norwich NR4 7UH, England. Insights into the structural and functional evolution of plant genomes afforded by the nucleotide sequences of chromosomes 2 and 4 of *Arabidopsis thaliana* (Review). *Yeast* **17:** (1) 1.

Barlow KL, Green J, Clewley JP*. 2000. *Cent Publ Hlth Lab, Div Virus Reference, 61 Colindale Ave, London NW9 5HT, England. Viral genome characterisation by the heteroduplex mobility and heteroduplex tracking assays (Review). *Rev Med Virol* **10:** (5) 321.

Brazma A, Vilo J. 2000. European Mol Biol Lab, European Bioinformat Inst, Hinxton Outstation, Cambridge CB10 1SD, England. Gene expression data analysis (Minireview). *FEBS Lett* **480:** (1) 17.

Califf RM. 2000. Duke Clin Res Inst, POB 17969, Durham, NC 27715, USA. Genetics, genomics, proteomics: What is a cardiologist to do?. *Am Heart J* **140:** (4) S48.

Cecconi F, Meyer BI. 2000. Max-Planck-Inst Biophys Chem, Dept Mol Cell Biol, Fassberg 11, DE-37077 Gottingen, Germany. Gene trap: A way to identify novel genes and unravel their biological function (Minireview). *FEBS Lett* **480:** (1) 63.

Celis JE, Kruhoffer M, Gromova I, Frederiksen C, Ostergaard M, Thykjaer T, Gromov P, Yu JS, Palsdottir H, Magnusson N, Orntoft TF. 2000. Aarhus Univ, Dept Med Biochem, Ole Worms Alle, Bldg 170, DK-8000 Aarhus C, Denmark. Gene expression profiling: Monitoring transcription and translation products using DNA microarrays and proteomics (Minireview). *FEBS Lett* **480:** (1) 2.

Chalmers MJ, Gaskell SJ. 2000. UMIST, Dept Chem, Michael Barber Ctr Mass Spectrometry, Manchester M60 1QD, England. Advances in mass spectrometry for proteome analysis. *Curr Opin Biotechnol* **11:** (4) 377.

Cho RJ, Campbell MJ. 2000. InGenuity Systems Inc, POB 2199, 2160 Gold St, 2nd Floor, Alviso, Ca 95002, USA. Transcription, genomes, function (Review). *Trends Genet* **16:** (9) 409.

Destenaves B, Thomas F. 2000. GENSET SA, Genomic Res Ctr, Dept Pharmacoeconom & Med Affairs, FR-91030 Evry, France. New advances in pharmacogenomics. *Curr Opin Chem Biol* **4:** (4) 440.

Dicks J. 2000. John Innes Ctr, Norwich Res Park, Colney, Norwich NR4 7UH, England. Graphical tools for comparative genome analysis (Review). *Yeast* **17:** (1) 6.

Dunwell JM. 2000. Univ Reading, Sch Plant Sci, Reading RG6 6AS, England. Crop genomics: Progress and prospects. *J Chem Technol Biotechnol* **75:** (10) 913.

Farkas G, Leibovitch BA, Elgin SCR*. 2000. *Washington Univ, Dept Biol, Campus Box 1229, 1 Brookings Dr, St Louis, Mo 63130, USA. Chromatin organization and transcriptional control of gene expression in *Drosophila* (Review). *Gene* **253:** (2) 117.

Fenyo D. 2000. ProteoMetrics LLC, 7 West 36th St, New York, NY 10018, USA. Identifying the proteome: Software tools (Review). *Curr Opin Biotechnol* **11:** (4) 391.

Glynne R, Ghandour G, Rayner J, Mack DH, Goodnow CC*. 2000. *Australian Natl Univ, John Curtin Sch Med Res, ACRF Genet Lab, Med Genome Ctr, POB 331, Canberra, ACT 2601, Australia. B-lymphocyte quiescence, tolerance and activation as viewed by global gene expression profiling on microarrays. *Immunol Rev* **176:** 216.

Goffeau A. 2000. Univ Catholique Louvain, Unite Biochim Physiol, Pl Croix Sud 2-20, BE-1348 Louvain, Belgium. Four years of post-genomic life with 6000 yeast genes (Minireview). *FEBS Lett* **480:** (1) 37.

Gromov PS, Celis JE. 2000. Univ Orhus, Inst Med Biochem, Orhus, Denmark. From genomics to proteomics. *Mol Biol-Engl Tr* **34:** (4) 508.

Gygi SP, Rist B, Aebersold R. 2000. Univ Washington, Dept Mol Biotechnol, Box 357730, Seattle, Wa 98195, USA. Measuring gene expression by quantitative proteome analysis. *Curr Opin Biotechnol* **11:** (4) 396.

Huynen M, Snel B, Lathe W, Bork P. 2000. European Mol Biol Lab, DE-69117 Heidelberg, Germany. Predicting protein function by genomic context: Quantitative evaluation and qualitative inferences. *Genome Res* **10:** (8) 1204.

Jordan B. 2000. Marseille Genopole, Case 901, Parc Sci de Luminy, FR-13288 Marseille 9, France. Forseeable futures for DNA chips (French). *M S-Med Sci* **16:** (8-9) 950.

Legrain P, Jestin JL, Schachter V. 2000. Hybrigenics, 180 Ave Daumesnil, FR-75012 Paris, France. From the analysis of protein complexes to proteome-wide linkage maps. *Curr Opin Biotechnol* **11:** (4) 402.

Legrain P, Selig L. 2000. Address as above. Genome-wide protein interaction maps using two-hybrid systems (Minireview). *FEBS Lett* **480:** (1) 32.

March R. 2000. AstraZeneca, Res & Dev Genet, Mereside, Alderley Park, Macclesfield SK10 4TG, England. Pharmacogenomics: The genomics of drug response (Review). *Yeast* **17:** (1) 16.

Meldrum D. 2000. Univ Washington, Dept Elect Engn, Genomation Lab, Seattle, Wa 98195, USA. Automation for genomics. Part I: Preparation for sequencing (Review). *Genome Res* **10:** (8) 1081.

Meldrum D. 2000. Address as above. Automation for genomics. Part II: Sequencers, microarrays and future trends (Review). *Genome Res* **10:** (9) 1288.

In order to keep subscribers up-to-date with the latest developments in their field, this current awareness service is provided by John Wiley & Sons and contains newly-published material on comparative and functional genomics. Each bibliography is divided into 16 sections. 1 Reviews & symposia; 2 General; 3 Large-scale sequencing and mapping; 4 Genome evolution; 5 Comparative genomics; 6 Pathways, gene families and regulons; 7 Pharmacogenomics; 8 Large-scale mutagenesis programmes; 9 Functional genomics; 10 Transcriptomics; 11 Proteomics; 12 Protein structural genomics; 13 Metabolomics; 14 Genomic approaches to development; 15 Technological advances; 16 Bioinformatics. Within each section, articles are listed in alphabetical order with respect to author. If, in the preceding period, no publications are located relevant to any one of these headings, that section will be omitted.

Copyright © 2001 John Wiley & Sons, Ltd.

Myler PJ, Stuart KD. 2000. Seattle Biomed Res Inst, 4 Nickerson St, Seattle, Wa 98109, USA. Recent developments from the *Leishmania* genome project. *Curr Opin Microbiol* **3:** (4) 412.

Paigen K, Eppig JT. 2000. Jackson Lab, 600 Main St, Bar Harbor, Me 04609, USA. A mouse phenome project (Review). *Mamm Genome* **11:** (9) 715.

Patterson SD. 2000. Amgen Inc, Biochem, MS 14-2-E, 1 Amgen Ctr Dr, Thousand Oaks, Ca 91320, USA. Proteomics: The industrialization of protein chemistry. *Curr Opin Biotechnol* **11:** (4) 413.

Reece RJ. 2000. Univ Manchester, Sch Biol Sci, Stopford Bldg, Oxford Rd, Manchester M13 9PT, England. Molecular basis of nutrient-controlled gene expression in *Saccharomyces cerevisiae*. *Cell Mol Life Sci* **57:** (8-9) 1161.

Riechmann JL, Ratcliffe OJ. 2000. Mendel Biotechnol, 21375 Cabot Blvd, Hayward, Ca 94545, USA. A genomic perspective on plant transcription factors. *Curr Opin Plant Biol* **3:** (5) 423.

Rockey DD, Lenart J, Stephens RS. 2000. Oregon State Univ, Dept Microbiol, Corvallis, Or 97331, USA. Genome sequencing and our understanding of chlamydiae (Mini-Review). *Infect Immun* **68:** (10) 5473.

Rubtsov PM. 2000. VA Engelhardt Mol Biol Inst, RU-117984 Moscow, Russia. Alternative promoters and RNA processing in expression of the eukaryotic genome. *Mol Biol-Engl Tr* **34:** (4) 535.

Sanders GHW, Manz A*. 2000. *Univ London, Imperial Coll Sci Technol & Med, Dept Chem, Zeneca SmithKline Beecham Ctr Analyt Sci, London SW7 1AZ, England. Chip-based microsystems for genomic and proteomic analysis. *Trends Anal Chem* **19:** (6) 364.

Sniegowski P. 1999. Univ Penn, Dept Biol, Philadelphia, Pa 19104, USA. The genomics of adaptation in yeast. *Curr Biol* **9:** (23) R897.

Springer PS. 2000. Univ Calif, Dept Bot & Plant Sci, Riverside, Ca 92521, USA. Gene traps: Tools for plant development and genomics. *Plant Cell* **12:** (7) 1007.

Tjalsma H, Bolhuis A, Jongbloed JDH, Bron S*, Van Dijl JM. 2000. *Groningen Biomol Sci & Biotechnol Int, Dept Genet, POB 14, NL-9750 AA Haren, The Netherlands. Signal peptide-dependent protein transport in *Bacillus subtilis*: A genome-based survey of the secretome. *Microbiol Mol Biol Rev* **64:** (3) 515.

Tsoka S, Ouzounis CA*. 2000. *EMBL, European Bioinformatics Inst, Computat Gen Grp, Res Programme, Cambridge Outstation, Cambridge CB10 1SD, England. Recent developments and future directions in computational genomics (Minireview). *FEBS Lett* **480:** (1) 42.

Turner M, Schuch W. 2000. Zeneca Agrochem, Jealotts Hill, Bracknell RG42 6ET, England. Post-transcriptional gene-silencing and RNA interference: Genetic immunity, mechanisms and applications (Review). *J Chem Technol Biotechnol* **75:** (10) 869.

Wixon J, Kell D. 2000. Univ Manchester, Sch Biol Sci, Stopford Bldg, Oxford Rd, Manchester M13 9PT, England. Website review: The Kyoto Encyclopedia of Genes and Genomes - KEGG. *Yeast* **17:** (1) 48.

Wixon J. 2000. Address as above. Meeting highlights: Plant and Animal Genome VIII and Agricultural Microbes Genome I - Town and Country Hotel, San Diego, California, USA, January 9-12 and 13-14 2000. *Yeast* **17:** (1) 56.

Zhouravleva GA, Mironova LN, Inge-Vechtomov SG. 2000. St Petersburg State Univ, Dept Genet & Breeding, RU-199034 St Petersburg, Russia. The yeast genome and the first steps toward the postgenomic era. *Mol Biol-Engl Tr* **34:** (4) 474.

Zucconi A, Panni S, Paoluzi S, Castagnoli L, Dente L, Cesareni G*. 2000. *Univ Roma Tor Vergata, Dept Biol Enrico Calef, via Ric Sci, IT-00133 Rome, Italy. Domain repertoires as a tool to derive protein recognition rules (Minireview). *FEBS Lett* **480:** (1) 49.

### 2 General

Albelda SM, Sheppard D. 2000. 421 Curie Blvd, Philadelphia, Pa 19104, USA. Functional genomics and expression profiling: Be there or be square. *Am J Respir Cell Mol Biol* **23:** (3) 265.

Bains W. 2000. Merlin Biosci, 12 St James's Sq, London SW1Y 4RB, England. The long-term value of genomics companies. *J Chem Technol Biotechnol* **75:** (10) 883.

Ben-Asher E, Chalifa-Caspi V, Horn-Saban S, Avidan N, Olender Z, Adato A, Glusman G, Safran M, Rubinstein M, Lancet D. 2000. Weizmann Inst Sci, Dept Mol Genet, IL-76100 Rehovot, Israel. Harvesting the human genome: The Israeli perspective. *Isr Med Assoc J* **2:** (9) 657.

Yates JR. 2000. Univ Washington, Dept Mol Biotechnol, Seattle, Wa 98195, USA. Mass spectrometry: From genomics to proteomics (Comment). *Trends Genet* **16:** (1) 5.

### 3 Large-scale sequencing and mapping

Altshuler D, Pollara VJ, Cowles CR, Van Etten WJ, Baldwin J, Linton L, Lander ES*. 2000. *MIT, Whitehead Inst, Ctr Genome Res, 9 Cambridge Ctr, Cambridge, Ma 02142, USA. An SNP map of the human genome generated by reduced representation shotgun sequencing. *Nature* **407:** (6803) 513.

Anderson SI, Lopez-Corrales NL, Gorick B, Archibald AL. 2000. Roslin Inst, Roslin EH25 9PS, Scotland. A large-fragment porcine genomic library resource in a BAC vector. *Mamm Genome* **11:** (9) 811.

Asamizu E, Nakamura Y, Sato S, Tabata S*. 2000. *Kazusa DNA Res Inst, 1532-3 Yana, Chiba 292 0812, Japan. A large scale analysis of cDNA in *Arabidopsis thaliana*: Generation of 12,028 non-redundant expressed sequence tags from normalized and site-selected cDNA libraries. *DNA Res* **7:** (3) 175.

Bawden AL, Glassberg KJ, Diggans J, Shaw R, Farmerie W, Moyer RW*. 2000. *Univ Florida, Coll Med, Dept Mol Genet & Microbiol, POB 100266, Gainesville, Fl 32610, USA. Complete genomic sequence of the *Amsacta moorei* entomopoxvirus: Analysis and comparison with other poxviruses. *Virology* **274:** (1) 120.

Blanc G, Barakat A, Guyot R, Cooke R*, Delseny I. 2000. *Univ Perpignan, Lab Genome & Dev Plantes, CNRS, Unite Mixte Rech 5096, FR-66860 Perpignan, France. Extensive duplication and reshuffling in the *Arabidopsis* genome. *Plant Cell* **12:** (7) 1093.

Blank WA, Stemke GW*. 2000. *Univ Alberta, Dept Biol Sci, Edmonton, Alberta, Canada T6G 2E9. A physical and genetic map of the *Mycoplasma hyopneumoniae* strain J genome. *Can J Microbiol* **46:** (9) 832.

Cameron RA, Mahairas G, Rast JP, Martinez P, Biondi TR, Swartzell S, Wallace JC, Poustka AJ, Livingston BT, Wray GA, Ettsohn CA, Lehrach H, Britten RJ, Davidson EH, Hood L. 2000. Calif Inst Technol, Div Biol, 156-29, Pasadena, Ca 91125, USA. A sea urchin genome project: Sequence scan, virtual map, and additional resources. *Proc Natl Acad Sci U S A* **97:** (17) 9514.

Clifford R, Edmonson M, Hu Y, Nguyen C, Scherpbier T, Buetow KH*. 2000. *NIH/NCI, Lab Populat Genet, Bethesda, Md 20892, USA. Expression-based genetic/physical maps of single-nucleotide polymorphisms identified by the cancer genome anatomy project. *Genome Res* **10:** (8) 1259.

Dandekar T, Huynen M, Ragula JT, Ueberle B, Zimmermann CU, Andrade MA, Doerks T, Sanchez-Pulido L, Snel B, Suyama M, Yuan YP, Herrmann R*, Bork P. 2000. *Campus Univ Autonoma, Ctr Nacl Biotecnol, ES-28049 Madrid, Spain. Re-annotating the *Mycoplasma pneumoniae* genome sequence: Adding value, function and reading frames. *Nucleic Acids Res* **28:** (17) 3278.

De Barros EG, Tingey S, Rafalski JA. 2000. Univ Fed Vicosa, Dipt Biol Geral, BIOAGRO, BR-36571-000 Vicosa, MG, Brazil. Sequence characterization of hypervariable regions in the soybean genome: Leucine-rich repeats and simple sequence repeats. *Genet Mol Biol* **23:** (2) 411.

Endrizzi MG, Hadinoto V, Growney JD, Miller W, Dietrich WF*. 2000. *Harvard Univ, Sch Med, Howard Hughes Med Inst, Boston, Ma 02115, USA. Genomic sequence analysis of the mouse Naip gene array. *Genome Res* **10:** (8) 1095.

Ffrench-Constant RH, Waterfield N, Burland V, Perna NT, Daborn PJ, Bowen D, Blattner FR. 2000. Univ Bath, Dept Biol & Biochem, South Bldg, Bath BA2 7AY, England. A genomic sample sequence of the entomopathogenic bacterium *Photorhabdus luminescens* W14: Potential

Copyright © 2001 John Wiley & Sons, Ltd. 

implications for virulence. *Appl Environ Microbiol* **66:** (8) 3310.

Frohme M, Camargo AA, Heber S, Czink C, Simpson AJD, Hoheisel JD, De Souza AP. 2000. Deutsch Krebsforschungszentrum, Funct Genome Anal, Neuenheimerfeld 506, DE-69120 Heidelberg, Germany. Mapping analysis of the *Xylella fastidiosa* genome. *Nucleic Acids Res* **28:** (16) 3100.

Heidelberg JF, Eisen JA, Nelson WC, Clayton RA, Gwinn ML, Dodson RJ, Haft DH, Hickey EK, Peterson JD, Umayam L *et al*. 2000. c/o Fraser CM, Inst Genomic Res, 9712 Med Ctr Dr, Rockville, Md 20850, USA. DNA sequence of both chromosomes of the cholera pathogen *Vibrio cholerae*. *Nature* **406:** (6795) 477.

Janssens W, Salminen MO, Laukkanen T, Heyndrickx L, Van der Auwera G, Colebunders R, McCutchan FE, Van der Groen G. 2000. Inst Trop Med, Dept Microbiol, Natl Str 155, BE-2000 Antwerp, Belgium. Near full-length genome analysis of HIV type 1 CRF02.AG subtype C and CRF02.AG subtype G recombinants. *AIDS Res Hum Retroviruses* **16:** (12) 1183.

Kuck U, Jekosch K, Holzamer P. 2000. Ruhr Univ Bochum, Lehrstuhl Allgemeine & Mol Bot, DE-44780 Bochum, Germany. DNA sequence analysis of the complete mitochondrial genome of the green alga *Scenedesmus obliquus*: Evidence for UAG being a leucine and UCA being a non-sense codon. *Gene* **253:** (1) 13.

McCarthy LC, Bihoreau MT, Kiguwa SL, Browne J, Watanabe TK, Hishigaki H, Tsuji A, Kiel S, Webber C, Davis ME *et al*. 2000. c/o James MR, Univ Oxford, Wellcome Trust Ctr Human Genet, Roosevelt Dr, Oxford OX3 7BN, England. A whole-genome radiation hybrid panel and framework map of the rat genome. *Mamm Genome* **11:** (9) 791.

Melkerson-Watson LJ, Rode CK, Zhang LX, Foxman B, Bloch CA. 2000. MSRB III, Room 8301, 1150 West Med Cr Dr, Ann Arbor, Mi 48109, USA. Integrated genomic map from uropathogenic *Escherichia coli* J96. *Infect Immun* **68:** (10) 5933.

Nagase T, Kikuno R, Nakayama M, Hirosawa M, Ohara O. 2000. Kazusa DNA Res Inst, 1532-3 Yana, Chiba 292 0812, Japan. Prediction of the coding sequences of unidentified human genes. XVIII. The complete sequences of 100 new cDNA clones from brain which code for large proteins *in vitro*. *DNA Res* **7:** (4) 273.

Porcel BM, Tran AN, Tammi M, Nyarady Z, Rydaker M, Urmenyu TP, Rondinelli E, Petterson U, Andersson B, Aslund L*. 2000. *Rudbeck Lab, Med Genet Sect, Dept Genet & Pathol, SE-75185 Uppsala, Sweden. Gene survey of the pathogenic protozoan *Trypanosoma cruzi*. *Genome Res* **10:** (8) 1103.

Ruepp A, Grami W, Santos-Martinez ML, Koretle KK, Volker C, Mewes HW, Frishman D, Stocker S, Lupas AN, Baumeister W*. 2000. *Max-Planck Inst Biochem, Klopferspitz 18A, DE-82152 Martinsried, Germany. The genome sequence of the thermoacidophilic scavenger *Thermoplasma acidophilum*. *Nature* **407:** (6803) 508.

Shigenobu S, Watanabe H, Hattori M, Sakaki Y*, Ishikawa H. 2000. *RIKEN, Genome Sci Ctr, Kitasato 1-15-1, Sagamihara, Kanagawa 228 855, Japan. Genome sequence of the endocellular bacterial symbiont of aphids *Buchnera* sp APS. *Nature* **407:** (6800) 81.

Siegel AF, Van den Engh G, Hood L, Trask B, Roach JC. 2000. Univ Washington, Dept Mol Biotechnol, Box 353200, Seattle, Wa 98195, USA. Modeling the feasibility of whole genome shotgun sequencing using a pairwise end strategy. *Genomics* **68:** (3) 237.

Souter A, McLean KJ, Smith WE, Munro AW*. 2000. *Univ Strathclyde, Royal Coll, Dept Pure & Appl Chem, 204 George St, Glasgow G1 1XW, Scotland. The genome sequence of *Mycobacterium tuberculosis* reveals cytochromes P450 as novel anti-TB drug targets. *J Chem Technol Biotechnol* **75:** (10) 933.

Steinmetz LM, Mindrinos M, Oefner PJ*. 2000. *Stanford Genome Technol Ctr, Palo Alto, Ca 94304, USA. Combining genome sequences and new technologies for dissecting the genetics of complex phenotypes. *Trends Plant Sci* **5:** (9) 397.

Stover CK, Pham XQ, Erwin AL, Mizoguchi SD, Warrener P, Hickey MJ, Brinkman FSL, Hufnagle WO, Kowalik DJ, Lagrou M *et al*. 2000. c/o Olson MV, Univ Washington, Genome Ctr, Dept Med & Genet, Box 352145, Seattle, Wa 98195, USA. Complete genome sequence of *Pseudomonas aeruginosa* PAO1, an opportunistic pathogen. *Nature* **406:** (6799) 959.

Tanno F, Nakatsu A, Toriyama S, Kojima M. 2000. Niigata Univ, Grad Sch Sci & Technol, Ikarashi 2-8050, Niigata 950 2181, Japan. Complete nucleotide sequence of Northern cereal mosaic virus and its genome organization. *Arch Virol* **145:** (7) 1373.

Yuan QP, Liang F, Hsiao J, Zismann V, Benito MI, Quackenbush J, Wing R, Buell R*. 2000. *Inst Genome Res, 9712 Med Ctr Dr, Rockville, Md 20850, USA. Anchoring of rice BAC clones to the rice genetic map *in silico*. *Nucleic Acids Res* **28:** (18) 3636.

### 4 Genome evolution

Hansmann S, Martin W*. 2000. *Univ Dusseldorf, Inst Bot III, Universitatsstr 1, DE-40225 Dusseldorf, Germany. Phylogeny of 33 ribosomal and six other proteins encoded in an ancient gene cluster that is conserved across prokaryotic genomes: Influence of excluding poorly alignable sites from analysis. *Int J Syst Evol Microbiol* **50:** (4) 1655.

Hurst LD, Randerson JP*. 2000. *Univ Bath, Dept Biol & Biochem, Claverton Down, Bath BA2 7AY, England. Dosage, deletions and dominance: Simple models of the evolution of gene expression. *J Theor Biol* **205:** (4) 641.

Korotkov EV, Korotkova MA, Rudenko VM. 2000. Russian Acad Sci, Ctr Bioengn, RU-117312 Moscow, Russia. MIR: Family of repeats common to vertebrate genomes. *Mol Biol-Engl Tr* **34:** (4) 468.

Kruglyak S, Durrett R*, Schug MD, Aquadro CF. 2000. *Cornell Univ, Dept Math, White Hall, Ithaca, NY 14853, USA. Distribution and abundance of microsatellites in the yeast genome can be explained by a balance between slippage events and point mutations. *Mol Biol Evol* **17:** (8) 1210.

Ladriere JM, Georis I, Guerineau M, Vandenhaute J. 2000. DSM Food Special, Screening & Strain Dev Lab, 15 rue Comtesses, FR-59472 Seclin, France. *Kluyveromyces marxianus* exhibits an ancestral *Saccharomyces cerevisiae* genome organization downstream of *ADH2*. *Gene* **255:** (1) 83.

Lo N, Tokuda G, Watanabe H, Rose H, Slaytor M, Maekawa K, Bandi C, Noda H. 1999. Univ Milan, Ist Patol Gen Vet, IT-20133 Milan, Italy. Evidence from multiple gene sequences indicates that termites evolved from wood-feeding cockroaches. *Curr Biol* **10:** (13) 801.

Scherbakov DV, Garber MB. 2000. Russian Acad Sci, Inst Prot Res, RU-142292 Pushchino, Russia. Overlapping genes in bacterial and phage genomes. *Mol Biol-Engl Tr* **34:** (4) 485.

Schmitz J, Ohme M, Zischler H. 2000. German Primate Ctr, Kellnerweg 4, DE-37077 Gottingen, Germany. The complete mitochondrial genome of *Tupaia belangeri* and the phylogenetic affiliation of Scandentia to other eutherian orders. *Mol Biol Evol* **17:** (9) 1334.

Snel B, Bork P, Huynen M. 2000. European Mol Biol Lab, Meyerhofstr 1, DE-69117 Heidelberg, Germany. Genome evolution: Gene fusion *versus* gene fission. *Trends Genet* **16:** (1) 9.

Tosto DS, Hopp HE. 2000. INTA Castelar, CICV-YA, Inst Biotecnol, CC 25, AR-1712 Castelar, Argentina. Suitability of AFLP markers for the study of genomic relationships within the *Oxalis tuberosa* alliance. *Plant Syst Evol* **223:** (3-4) 201.

Vrati S. 2000. Natl Inst Immunol, Virol Lab, Aruna Asaf Ali Marg, JNU Complex, IN-110067 New Delhi, India. Comparison of the genome sequences and the phylogenetic analyses of the GP78 and the Vellore P20778 isolates of Japanese encephalitis virus from India. *J Biosci* **25:** (3) 257.

Yang FT, Graphodatsky AS, O'Brien PCM, Colabella A, Solanky N, Squire M, Sargan DR, Ferguson-Smith MA*. 2000. *Univ Cambridge, Dept Clin Vet Med, Ctr Vet Sci, Madingley Rd, Cambridge CB3 0ES, England. Reciprocal chromosome painting illuminates the history of genome evolution of the domestic cat, dog and human. *Chromosome Res* **8:** (5) 393.

### 5 Comparative genomics

Acarkan A, Rossberg M, Koch M, Schmidt R*. 2000. *Max-Planck-Gesell, Max Delbruck Lab, Carl von Linne Weg 10, DE-50829 Co-

Copyright © 2001 John Wiley & Sons, Ltd. 

logne, Germany. Comparative genome analysis reveals extensive conservation of genome organisation for *Arabidopsis thaliana* and *Capsella rubella*. *Plant J* **23:** (1) 55.

Band MR, Larson JH, Rebeiz M, Green CA, Heyen DW, Donovan J, Windish R, Steining C, Mahyuddin P, Womack JE, Lewin HA*. 2000. *Univ Illinois, Dept Anim Sci, Urbana, Il 61801, USA. An ordered comparative map of the cattle and human genomes. *Genome Res* **10:** (9) 1359.

Barbazuk WB, Korf I, Kadavi C, Heyen J, Tate S, Wun E, Bedell JA, McPherson JD, Johnson SL*. 2000. *Washington Univ, Sch Med, Dept Genet, St Louis, Mo 63130, USA. The syntenic relationship of the zebrafish and human genomes. *Genome Res* **10:** (9) 1351.

Bennetzen JL. 2000. Purdue Univ, Dept Biol Sci, West Lafayette, In 47907, USA. Comparative sequence analysis of plant nuclear genomes: Microcolinearity and its many exceptions. *Plant Cell* **12:** (7) 1021.

Forozan F, Mahlamaki EH, Monni O, Chen YD, Veldman R, Jiang Y, Gooden GC, Ethier SP, Kallioniemi A, Kallioniemi OP*. 2000. *NIH/NHGRI, Canc Genet Branch, Bldg 49, Room 4A24, 49 Convent Dr, MSC 4470, Bethesda, Md 20892, USA. Comparative genomic hybridization analysis of 38 breast cancer cell lines: A basis for interpreting complementary DNA microarray data. *Cancer Res* **60:** (16) 4519.

Goureau A, Vignoles M, Pinton P, Gellin J, Yerle M. 2000. INRA, Lab Genet Cellulaire, BP27, FR-31326 Castanet Tolosan, France. Improvement of comparative map between porcine chromosomes 1 and 7 and human chromosomes 6, 14, and 15 by using humans YACs. *Mamm Genome* **11:** (9) 796.

Grigoriev A. 2000. GPC Biotech, Fraunhoferstr 20, Martinsried, Germany. Graphical genome comparison: Rearrangements and replication origin of *Helicobacter pylori*. *Trends Genet* **16:** (9) 376.

Kent WJ, Zahler AM. 2000. Univ Calif, Dept Biol, Santa Cruz, Ca 95064, USA. Conservation, regulation, synteny, and introns in a large-scale *C. briggsae-C. elegans* genomic alignment. *Genome Res* **10:** (8) 1115.

Ku HM, Vision T, Liu JP, Tanksley SD*. 2000. *Cornell Univ, Dept Plant Breeding, Ithaca, NY 14853, USA. Comparing sequenced segments of the tomato and *Arabidopsis* genomes: Large-scale duplication followed by selective gene loss creates a network of synteny. *Proc Natl Acad Sci U S A* **97:** (16) 9121.

Larkin DM, Serov OL, Borodin PM, Zhdanova NS, Searle JB. 2000. Russian Acad Sci, Inst Cytol & Genet, RU-630090 Novosibirsk, Russia. Comparative genome mapping in mammals: The shrew map. *Acta Theriol* **45:** (Suppl 1) 131.

McLysaght A, Enright AJ, Skrabanek L, Wolfe KH*. 2000. *Univ Dublin, Trinity Coll, Dept Genet, Dublin 2, Rep Ireland. Estimation of synteny conservation and genome compaction between pufferfish (Fugu) and human. *Yeast* **17:** (1) 22.

Muniyappa K, Vaze MB, Ganesh N, Reddy MS, Guhan N, Venkatesh R. 2000. Indian Inst Sci, Dept Biochem, IN-560012 Bangalore, India. Comparative genomics of *Mycobacterium tuberculosis* and *Escherichia coli* for recombination (*rec*) genes. *Microbiology* **146:** (9) 2093.

O'Neill CM, Bancroft I*. 2000. *John Innes Ctr Plant Sci Res, Dept Brassica & Oilseeds Res, Norwich Res Pk, Norwich NR4 7UH, England. Comparative physical mapping of segments of the genome of *Brassica oleracea* var. *alboglabra* that are homoeologous to sequenced regions of chromosomes 4 and 5 of *Arabidoposis thaliana*. *Plant J* **23:** (2) 233.

Paulsen IT, Nguyen L, Sliwinski MK, Rabus R, Saier MH. 2000. Inst Genomic Res, 9712 Med Ctr Dr, Rockville, Md 20850, USA. Microbial genome analyses: Comparative transport capabilities in eighteen prokaryotes. *J Mol Biol* **301:** (1) 75.

Tan SH, Nishiguchi M, Murata M, Motoyoshi F*. 2000. *Univ Putra, Fac Food Sci & Biotechnol, Serdang, Malaysia. The genome structure of kyuri green mottle mosaic tobamovirus and its comparison with that of cucumber green mottle mosaic tobamovirus. *Arch Virol* **145:** (6) 1067.

Voigt B, Kitada K, Kloting I, Serikawa T. 2000. Kyoto Univ, Inst Lab Anim, Grad Sch Med, Kyoto 606 8501, Japan. Genetic comparison between laboratory rats and Japanese and German wild rats. *Mamm Genome* **11:** (9) 789.

Wozniak G, Trontelj P, Rupnik M*. 2000. *Univ Ljubljana, Biotech Fac, Dept Biol, Vecna pot 111, SI-1000 Ljubljana, Slovenia. Genomic relatedness of *Clostridium difficile* strains from different toxinotypes and serogroups. *Anaerobe* **6:** (4) 261.

Zabarovsky ER. 2000. VA Engelhardt Mol Biol Inst, RU-117984 Moscow, Russia. Novel strategies to clone identical and distinct DNA sequences for several complex genomes. *Mol Biol-Engl Tr* **34:** (4) 521.

### 6 Pathways, gene families and regulons

Antelmann H, Scharf C, Hecker M*. 2000. *Univ Greifswald, Inst Mikrobiol, DE-17487 Greifswald, Germany. Phosphate starvation-inducible proteins of *Bacillus subtilis*: Proteomics and transcriptional analysis. *J Bacteriol* **182:** (16) 4478.

Kaebernick M, Neilan BA*, Borner T, Dittmann E. 2000. *Univ New Sth Wales, Sch Microbiol & Immunol, Sydney, NSW 2052, Australia. Light and the transcriptional response of the microcystin biosynthesis gene cluster. *Appl Environ Microbiol* **66:** (8) 3387.

Kubo M, Kakimoto T*. 2000. *Osaka Univ, Grad Sch Sci, Dept Biol, Osaka 560 0043, Japan. The *CYTOKININ-HYPERSENSITIVE* genes of *Arabidopsis* negatively regulate the cytokinin-signaling pathway for cell division and chloroplast development. *Plant J* **23:** (3) 385.

Nadimpalli R, Yalpani N, Johal GS, Simmons CR*. 2000. *Pioneer Hi-Bred Int Inc, Bioinformat Dept, 7250 NW 62nd Ave, Johnston, Ia 50131, USA. Prohibitins, stomatins, and plant disease response genes compose a protein superfamily that controls cell proliferation, ion channel regulation, and death. *J Biol Chem* **275:** (38) 29579.

Poggio S, Aguilar C, Osorio A, Gonzalez-Pedrajo B, Dreyfus G, Camarena L*. 2000. *Univ Nacl Autonoma Mexico, Inst Invest Biomed, Dept Biol Mol, Apdo Postal 70-228, MX-04510 Mexico City, DF, Mexico. $\sigma^{54}$ Promoters control expression of genes encoding the hook and basal body complex in *Rhodobacter sphaeroides*. *J Bacteriol* **182:** (20) 5787.

Thomas G, Coutts G, Merrick M. 2000. John Innes Ctr Plant Sci Res, Dept Mol Microbiol, Colney Lane, Norwich NR4 7UH, England. The *glnKamtB* operon: A conserved gene pair in prokaryotes. *Trends Genet* **16:** (1) 11.

Vitreshchak AG, Gelfand MS. 2000. Russian Acad Sci, Inst Problems Data Transmission, RU-101447 Moscow, Russia. Computer analysis of control signals in bacterial genomes: Attenuators of operons of aromatic amino acids metabolism. *Mol Biol-Engl Tr* **34:** (4) 461.

### 7 Pharmacogenomics

Cacabelos R, Alvarez A, Lombardi A, Fernandez-Novoa L, Corzo L, Perez P, Laredo M, Pichel V, Hernandez A, Varela M, Figueroa J, Prous J, Windisch M, Vigo C. 2000. Inst CNS Disorders, EuroEspes Biomed Res Ctr, ES-15166 La Coruna, Spain. Pharmacological treatment of Alzheimer disease: From psychotropic drugs and cholinesterase inhibitors to pharmacogenomics. *Drugs Today* **36:** (7) 415.

Jenson JS, Childerstone A, Takamatsu HH, Dixon LK, Parkhouse RME. 2000. Univ Glasgow, Dept Vet Parasitol, Bearsden Rd, Glasgow G61 1QH, Scotland. The cellular immune recognition of proteins expressed by an African swine fever virus random genomic library. *J Immunol Methods* **242:** (1-2) 33.

Loging WT, Lal A, Siu IM, Loney TL, Wikstrand CJ, Marra MA, Prange C, Bigner DD, Strausberg RL, Riggins GJ*. 2000. *Duke Univ, Med Ctr, Durham, NC 27710, USA. Identifying potential tumor markers and antigens by database mining and rapid expression screening. *Genome Res* **10:** (9) 1393.

Mei R, Galipeau PC, Prass C, Berno A, Ghandour G, Patil N, Wolff RK, Chee MS, Reid BJ, Lockhart DJ. 2000. Affymetrix Inc, Santa Clara, Ca 95051, USA. Genome-wide detection of allelic imbalance using human SNPs and high-density DNA arrays. *Genome Res* **10:** (8) 1126.

Melby PC, Ogden GB, Flores HA, Zhao WG, Geldmacher C, Biediger NM, Ahuja SK, Uranga J, Melendez M. 2000. Univ Texas, Hlth Sci

Copyright © 2001 John Wiley & Sons, Ltd. 

Ctr, Department Med, Div Infect Dis, 7703 Floyd Curl Dr, Mailstop 7881, San Antonio, Tx 78229, USA. Identification of vaccine candidates for experimental visceral leishmaniasis by immunization with sequential fractions of a cDNA expression library. *Infect Immun* **68:** (10) 5595.

### 8 Large-scale mutagenesis programmes

De Angelis MH, Flaswinkel H, Fuchs H, Rathkolb B, Soewarto D, Marschall S, Heffner S, Pargent W, Wuensch K, Jung M *et al*. 2000. GSF Munich, Res Ctr Environm & Hlth, Inst Expt Genet, Neuherberg, Germany. Genome-wide, large-scale production of mutant mice by ENU mutagenesis. *Nat Genet* **25:** (4) 444.

Gehring AM, Nodwell JR, Beverley SM, Losick R*. 2000. *Harvard Univ, Dept Mol & Cellular Biol, 16 Divin Ave, Cambridge, Ma 02138, USA. Genomewide insertional mutagenesis in *Streptomyces coelicolor* reveals additional genes involved in morphological differentiation. *Proc Natl Acad Sci U S A* **97:** (17) 9642.

Kennerdell JR, Carthew RW*. 2000. *Univ Pittsburgh, Dept Biol Sci, 4249 5th Ave, 217 Clapp Hall, Pittsburgh, Pa 15260, USA. Heritable gene silencing in *Drosophila* using double-stranded RNA. *Nat Biotechnol* **18:** (8) 896.

Nolan PM, Peters J, Strivens M, Rogers D, Hagan J, Spurr N, Gray IC, Vizor L, Brooker D, Whitehill E *et al*. 2000. c/o Brown SDM, MRC Mammalian Genet Unit, Harwell, England. A systematic, genome-wide, phenotype-driven mutagenesis programme for gene function studies in the mouse. *Nat Genet* **25:** (4) 440.

### 9 Functional genomics

Bolton AJ, Woods DE*. 2000. *Univ Calgary, Dept Microbiol & Infect Dis, Hlth Sci Ctr, 3330 Hosp Dr, Calgary, Alberta, Canada T2N 4N1. Self-cloning minitransposon *phoA* gene-fusion system promotes the rapid genetic analysis of secreted proteins in Gram-negative bacteria. *Biotechniques* **29:** (3) 470.

Delneri D, Tomlin GC, Wixon JL, Hutter A, Sefton M, Louis EJ, Oliver SG*. 2000. *Univ Manchester, Sch Biol Sci, 2-205 Stopford Bldg, Oxford Rd, Manchester M13 9PT, England. Exploring redundancy in the yeast genome: An improved strategy for use of the cre-*lox*P system. *Gene* **252:** (1-2) 127.

Ermolaeva MD, Khalak HG, White O, Smith HO, Salzberg SL. 2000. Inst Genomic Res, 9712 Med Ctr Dr, Rockville, Md 20850, USA. Prediction of transcription terminators in bacterial genomes. *J Mol Biol* **301:** (1) 27.

Kong HM, Lin LF, Porter N, Stickel S, Byrd D, Posfai J, Roberts RJ. 2000. New England Biolabs Inc, 32 Tozer Rd, Beverly, Ma 01915, USA. Functional analysis of putative restriction-modification system genes in the *Helicobacter pylori* J99 genome. *Nucleic Acids Res* **28:** (17) 3216.

Pace T, Scotti R, Janse CJ, Waters AP, Birago C, Ponzi M*. 2000. *Ist Super Sanita, Lab Biol Cellulare, Viale Regina Elena 299, IT-00161 Rome, Italy. Targeted terminal deletions as a tool for functional genomics studies in *Plasmodium*. *Genome Res* **10:** (9) 1414.

Porcel BM, Aslund L, Pettersson U, Andersson B*. 2000. *Rudbeck Lab, Dept Genet & Pathol, Med Genet Sect, SE-75185 Uppsala, Sweden. *Trypanosoma cruzi*: A putative vacuolar ATP synthase subunit and a CAAX prenyl protease-encoding gene, as examples of gene identification in genome projects. *Exp Parasitol* **95:** (3) 176.

Tyagi AK, Mohanty A. 2000. Univ Delhi, Ctr Plant Mol Biol, Sth Campus, Benito Juarez Rd, IN-110021 New Delhi, India. Rice transformation for crop improvement and functional genomics. *Plant Sci* **158:** (1-2) 1.

### 10 Transcriptomics

Ahr A, Holtrich U, Karn T, Solbach C, Gatje R, Scharl A, Strebhardt K, Kaufmann M. 2000. Univ Frankfurt, Frauenklin, Bereich Mol Gynakol & Geburtshilfe, Theodor Stern Kai 7, DE-60590 Frankfurt, Germany. Detection of differentially expressed genes in breast cancer with cDNA-array hybridization (German, English Abstract). *Geburtshilfliche Frauenheilk* **60:** (8) 412.

Anderson KM, Alrefai WA, Bonomi PA, Anderson CA, Dudeja P, Harris JE. 2000. Rush Med Coll, Dept Med, 1830 West Harrison, Chicago, Il 60612, USA. A genomic response of H-358 bronchiolar carcinoma cells to MK 886, an inhibitor of 5-lipoxygenase, assessed with a cDNA array. *Anticancer Res* **20:** (4) 2433.

Arfin SM, Long AD, Ito ET, Tolleri L, Riehle MM, Paegle ES, Hatfield GW. 2000. Univ Calif, Coll Med, Dept Biol Chem, Irvine, Ca 92697, USA. Global gene expression profiling in *Escherichia coli* K12: The effects of integration host factor. *J Biol Chem* **275:** (38) 29672.

Bittner M, Meitzer P, Chen Y, Jiang Y, Seftor E, Hendrix M, Radmacher M, Simon R, Yakhini Z, Ben Dor A *et al*. 2000. NIH/Natl Human Genome Res Inst, Canc Genet Branch, Bethesda, Md 20892, USA. Molecular classification of cutaneous malignant melanoma by gene expression profiling. *Nature* **406:** (6795) 536.

Brocklehurst KR, Morby AP. 2000. Cardiff Univ, Sch Biosci, Museum Ave, Cardiff CF10 3US, Wales. Metal-ion tolerance in *Escherichia coli*: Analysis of transcriptional profiles by gene-array technology. *Microbiology* **146:** (9) 2277.

Eickhoff H, Schuchhardt J, Ivanov I, Meier-Ewert S, O'Brien J, Malik A, Tandon N, Wolski EW, Rohlfs E, Nyarsik L, Reinhardt R, Nietfield W, Lehrach H. 2000. Max Planck Inst Mol Genet, Ihnestr 73, DE-14195 Berlin, Germany. Tissue gene expression analysis using arrayed normalized cDNA libraries. *Genome Res* **10:** (8) 1230.

Endo M, Kokubun T, Takahata Y, Higashitani A, Tabata S, Watanabe M*. 2000. *Iwate Univ, Fac Agr, Lab Plant Breeding, 3-18-8 Ueda, Morioka, Iwate 020 8550, Japan. Analysis of expressed sequence tags of flower buds in *Lotus japonicus*. *DNA Res* **7:** (3) 213.

Fidock DA, Nguyen TV, Ribeiro JM, Valenzuela JC, James AA. 2000. Yeshiva Univ Albert Einstein Coll Med, Dept Microbiol & Immunol, 1300 Morris Pk Ave, Bronx, NY 10461, USA. *Plasmodium falciparum:* Generation of a cDNA library enriched in sporozolte-specific transcripts by directional tag subtractive hybridization. *Exp Parasitol* **95:** (3) 220.

Gabrielsson BL, Carlsson B, Carlsson LMS*. 2000. *Univ Gothenburg, Sahlgrens Univ Hosp, Dept Internal Med, RCEM, Grona Straket 8, SE-41345 Gothenburg, Sweden. Partial genome scale analysis of gene expression in human adipose tissue using DNA array. *Obes Res* **8:** (5) 374.

He ZW, Ren CP, Xu LG, Liu WD, Xie L, Li ZK, Yao KT*. 2000. *Hunan Med Univ, Chinese Minist Hlth, Kay Lab Carcinogenesis, CN-410078 Changsha, Peoples Rep China. Profiling gene expression patterns of nasopharyngeal carcinoma and normal nasopharynx tissues with cDNA microarray. *Chin Sci Bull* **45:** (9) 830.

Hong TM, Yang PC*, Peck K, Chen JJW, Yang SC, Chen YC, Wu CW. 2000. *Natl Taiwan Univ Hosp, Dept Internal Med, 7 Chung Shan Sth Rd, Taipei 100, Taiwan. Profiling the downstream genes of tumor suppressor *PTEN* in lung cancer cells by complementary DNA microarray. *Am J Respir Cell Mol Biol* **23:** (3) 355.

Ichikawa JK, Norris A, Bangera MG, Geiss GK, Van Wout AB, Bumgarner RE, Lory S*. 2000. *Harvard Univ, Sch Med, Boston, Ma 02115, USA. Interaction of *Pseudomonas aeruginosa* with epithelial cells: Identification of differentially regulated genes by expression microarray analysis of human cDNAs. *Proc Natl Acad Sci U S A* **97:** (17) 9659.

Jia MH, Larossa RA, Lee JM, Rafalski A, Derose E, Gonye G, Xue ZX*. 2000. *DuPont Co Inc, Cent Res, Expt Stn, POB 80173, Rt 141 & Rising Sun Lane, Wilmington, De 19880, USA. Global expression profiling of yeast treated with an inhibitor of amino acid biosynthesis, sulfometuron methyl. *Physiol Genomics* **3:** (2) 83.

Kudoh K, Ramanna M, Ravatn R, Elkahloun AG, Bittner ML, Meltzer PS, Trent JM, Dalton WS, Chin KV*. 2000. *Univ Med & Dent New Jersey, Robert Wood Johnson Med Sch, Canc Inst, 195 Little Albany St, New Brunswick, NJ 08901, USA. Monitoring the expression profiles of doxorubicin-induced and doxorubicin-resistant cancer cells by cDNA microarray. *Cancer Res* **60:** (15) 4161.

Lee MLT, Kuo FC, Whitmore GA, Sklar J. 2000. Brigham & Women's

Copyright © 2001 John Wiley & Sons, Ltd. 

Hosp, Channing Lab, HMS, Dept Med, 181 Longwood Ave, Boston, Ma 02115, USA. Importance of replication in microarray gene expression studies: Statistical methods and evidence from repetitive cDNA hybridizations. *Proc Natl Acad Sci U S A* **97:** (18) 9834.

Liang F, Holt I, Pertea G, Karamycheva S, Salzberg SL, Quackenbush J*. 2000. *Inst Genomic Res, 9712 Med Ctr Dr, Rockville, Md 20850, USA. An optimized protocol for analysis of EST sequences. *Nucleic Acids Res* **28:** (18) 3657.

Livesey FJ, Furukawa T, Steffen MA, Church GM, Cepko CL*. 1999. *Harvard Univ, Sch Med, Dept Genet, 200 Longwood Ave, Boston, Ma 02115, USA. Microarray analysis of the transcriptional network controlled by the photoreceptor homeobox gene *Crx*. *Curr Biol* **10:** (6) 301.

Lombardfa LJ, Cadahia-Rodriguez JL, Freire-Picos MA, Gonzalez-Siso MI, Rodriguez-Torres AM, Cerdan ME*. 2000. *Univ La Coruna, Dept Biol Celular & Mol, Campus Zapyateira s/n, ES-15071 La Coruna, Spain. Transcript analysis of 203 novel genes from *Saccharomyces cerevisiae* in *hap1* and *rox1* mutant backgrounds. *Genome* **43:** (5) 881.

Lopez MC, Baker HV*. 2000. *University Florida, College Medicine, Dept Mol Genet & Microbiol, Box 100266, Gainesville, Fl 32610, USA. Understanding the growth phenotype of the yeast *gcr1* mutant in terms of global genomic expression patterns. *J Bacteriol* **182:** (17) 4970.

McDonagh PD, Myler PJ, Stuart K*. 2000. *Seattle Biomed Res Inst, 4 Nickerson St, Seattle, Wa 98109, USA. The unusual gene organization of *Leishmania major* chromosome 1 may reflect novel transcription processes. *Nucleic Acids Res* **28:** (14) 2800.

Nakachi N, Matsunaga K, Klein TW, Friedman H, Yamamoto Y*. 2000. *Univ Sth Florida, Coll Med, Dept Med Microbiol & Immunol, 12901 Bruce B Downs Blvd, Tampa, Fl 33612, USA. Differential effects of virulent *versus* avirulent *Legionella pneumophila* on chemokine gene expression in murine alveolar macrophages determined by cDNA expression array technique. *Infect Immun* **68:** (10) 6069.

Nikaido I, Asamizu E, Nakajima M, Nakamura Y, Saga N, Tabata S*. 2000. *Kazusa DNA Res Inst, Chiba 292 0812, Japan. Generation of 10,154 expressed sequence tags from a leafy gametophyte of a marine red alga, *Porphyra yezoensis*. *DNA Res* **7:** (3) 223.

Peek RM, Van Doorn LJ, Donahue JP, Tham KT, Figueiredo C, Blaser MJ, Miller GG. 2000. Vanderbilt University, School Med, Dept Med, Div Gastroenterol, 1161 21st Ave Sth, Med Ctr Nth, Nashville, Tn 37232, USA. Quantitative detection of *Helicobacter pylori* gene expression *in vivo* and relationship to gastric pathology. *Infect Immun* **68:** (10) 5488.

Steimer A, Amedeo P, Afsar K, Fransz P, Scheid OM, Paszkowski J. 2000. Friedrich Miescher Inst, Postfach 2543, CH-4002 Basel, Switzerland. Endogenous targets of transcriptional gene silencing in *Arabidopsis*. *Plant Cell* **12:** (7) 1165.

Taylor LA, Carthy CM, Yang DC, Saad K, Wong D, Schreiner G, Stanton LW, McManus BM*. 2000. *Univ British Columbia, St Paul's Hosp, Dept Pathol & Lab Med, 1081 Burrard St, Vancouver, Brit Columbia, Canada V6Z 1Y6. Host gene regulation during coxsackievirus B3 infection in mice: Assessment by microarrays. *Circ Res* **87:** (4) 328.

Wang RC, Guegler K, La Brie ST, Crawford NM*. 2000. *Univ Calif San Diego, Div Biol, La Jolla, Ca 92093, USA. Genomic analysis of a nutrient response in *Arabidopsis* reveals diverse expression patterns and novel metabolic and potential regulatory genes induced by nitrate. *Plant Cell* **12:** (8) 1491.

Ye RW, Tao W, Bedzyk L, Young T, Chen M, Li L. 2000. DuPont Co Inc, Cent Res & Dev, Expt Stn, Route 141 & Henry Clay Rd, Wilmington, De 19880, USA. Global gene expression profiles of *Bacillus subtilis* grown under anaerobic conditions. *J Bacteriol* **182:** (16) 4458.

Yoshikawa T, Nagasugi Y, Azuma T, Kato M, Sugano S, Hashimoto K, Masuho Y, Muramatsu M, Seki N*. 2000. *Helix Res Inst, Biol Technol Lab, 1532-3 Yana, Chiba 292 0812, Japan. Isolation of novel mouse genes differentially expressed in brain using cDNA microarray. *Biochem Biophys Res Commun* **275:** (2) 532.

### 11 Proteomics

Balasubramanian S, Schneider T, Gerstein M, Regan L*. 2000. *Yale Univ, Dept Mol Biophys & Biochem, 266 Whitney Ave, New Haven, Ct 06520, USA. Proteomics of *Mycoplasma genitalium*: Identification and characterization of unannotated and atypical proteins in a small model genome. *Nucleic Acids Res* **28:** (16) 3075.

Barrett J, Jefferies JR, Brophy PM. 2000. Univ Wales, Inst Biol Sci, Aberystwyth SY23 3DA, Wales. Parasite proteomics. *Parasitol Today* **16:** (9) 400.

Brancia FL, Oliver SG, Gaskell SJ*. 2000. *UMIST, Michael Barber Ctr Mass Spectrom, PO Box 88, Manchester M60 1QD, England. Improved matrix-assisted laser desorption/ionization mass spectrometric analysis of tryptic hydrolysates of proteins following guanidination of lysine-containing peptides. *Rapid Commun Mass Spectrom* **14:** (21) 2070.

Gygi SP, Corthals GL, Zhang Y, Rochon Y, Aebersold R*. 2000. *Univ Washington, Dept Mol Biotechnol, POB 357730, Seattle, Wa 98195, USA. Evaluation of two-dimensional gel electrophoresis-based proteome analysis technology. *Proc Natl Acad Sci U S A* **97:** (17) 9390.

Ji JY, Chakraborty A, Geng M, Zhang X, Amini A, Bina M, Regnier F*. 2000. *Purdue Univ, Dept Chem, West Lafayette, In 47907, USA. Strategy for qualitative and quantitative analysis in proteomics based on signature peptides. *J Chromatogr B* **745:** (1) 197.

Kristensen DB, Kawada N, Imamura K, Miyamoto Y, Tateno C, Seki S, Kuroki T, Yoshizato K*. 2000. *Hiroshima Univ, Grad Sch Sci, Dept Biol Sci, Dev Biol Lab, 1-3-1 Kagamiyama, Hiroshima 739 8526, Japan. Proteome analysis of rat hepatic stellate cells. *Hepatology* **32:** (2) 268.

McKerrow JH, Bhargava V, Hansell E, Huling S, Kuwahara T, Matley M, Coussens L, Warren R. 2000. Univ Calif, VA Med Ctr, Dept Pathol, 4150 Clement St, San Francisco, Ca 94121, USA. A functional proteomics screen of proteases in colorectal carcinoma. *Mol Med* **6:** (5) 450.

Natera SHA, Guerreiro N, Djordjevic NA*. 2000. *Australian Natl Univ, Res Sch Biol Sci, Plant Microbe Interact Grp, POB 475, Canberra, ACT 2601, Australia. Proteome analysis of differentially displayed proteins as a tool for the investigation of symbiosis. *Mol Plant Microbe Interact* **13:** (9) 995.

Siegel RW, Allen B, Pavlik P, Marks JD, Bradbury A*. 2000. *Los Alamos Natl Lab, Biosci Div, POB 1663, Los Alamos, NM 87545, USA. Mass spectral analysis of a protein complex using single-chain antibodies selected on a peptide target: Applications to functional genomics. *J Mol Biol* **302:** (2) 285.

Yu LR, Wang N, Wu G, Xu YH, Xia QC*. 2000. *Chinese Acad Sci, Inst Biochem, CN-200031 Shanghai, Peoples Rep China. Two-dimensional gel electrophoresis analysis of the proteomes expressed in the human hepatoma cell line BEL-7404 and normal liver cell line L-02. *Chin Sci Bull* **45:** (12) 1113.

Yu LR, Zeng R, Shao XX, Wang N, Xu YH, Xia QC*. 2000. *Chinese Acad Sci, Inst Biochem, 320 Yue Yang Rd, CN-200031 Shanghai, Peoples Rep China. Identification of differentially expressed proteins between human hepatoma and normal liver cell lines by two-dimensional electrophoresis and liquid chromatography-ion trap mass spectrometry. *Electrophoresis* **21:** (14) 3058.

### 12 Protein structural genomics

Hirayama K. 2000. Ajinomoto Co Inc, Cent Res Lab, 1-1 Suzuki-cho, Kawasaki-ku, Kawasaki 210 8681, Japan. Study of the mechanism of biological systems using mass spectrometry: From proteome analysis to structural biology. *J Mass Spectrom Soc Jpn* **48:** (5) 289.

### 13 Metabolomics

Lindon JC, Nicholson JK, Holmes E, Everett JR. 2000. Univ London, Imperial Coll Sci Technol & Med, Div Biomed Sci, Sir Alexander

Copyright © 2001 John Wiley & Sons, Ltd. 

Fleming Bldg, London SW7 1AZ, England. Metabonomics: Metabolic processes studied by NMR spectroscopy of biofluids. *Concept Magn Reson* **12:** (5) 289.

### 14 Genomic approaches to development

Cairney J, Xu NF, Mackay J, Pullman J. 2000. Inst Paper Sci & Technol, Forest Biol Grp, 500 10th St, Atlanta, Ga 30318, USA. Transcript profiling: A tool to assess the development of conifer embryos. *In vitro Cell Dev Biol-Plant* **36:** (3) 155.

Guo DC, Wu Y, Kaplan HB*. 2000. *Univ Texas, Sch Med, Dept Microbiol & Mol Genet, 6431 Fannin, Houston, Tx 77030, USA. Identification and characterization of genes required for early *Myxococcus xanthus* developmental gene expression. *J Bacteriol* **182:** (16) 4564.

Kelsh RN, Schmid B, Eisen JS. 2000. Max-Planck-Inst Entwicklungsbiol, Abt 3, Spemannstr 35, DE-72076 Tubingen, Germany. Genetic analysis of melanophore development in zebrafish embryos. *Dev Biol* **225:** (2) 277.

Petkov PM, Kim K, Sandhu J, Shafritz DA, Dabeva MD*. 2000. *Yeshiva Univ, Albert Einstein Coll Med, Marion Bessin Liver Res Ctr, 1300 Morris Pk Ave, Bronx, NY 10461, USA. Identification of differentially expressed genes in epithelial stem/progenitor cells of fetal rat liver. *Genomics* **68:** (2) 197.

Popovici RM, Kao LC, Giudice LC. 2000. Stanford Univ, Med Ctr, Dept Obstet & Gynaecol, Div Reprod Endocrinol & Infertil, Stanford, Ca 94305, USA. Discovery of new inducible genes in *in vitro* decidualized human endometrial stromal cells using microarray technology. *Endocrinology* **141:** (9) 3510.

Tanaka TS, Jaradat SA, Lim MK, Kargul GJ, Wang XH, Grahovac MJ, Pantano S, Sano Y, Piao Y, Nagaraja R, Doi H, Wood WH, Becker KG, Ko MSH*. 2000. *NIH/NIA, Genet Lab, 333 Cassell Dr, Suite 4000, Baltimore, Md 21224, USA. Genome-wide expression profiling of mid-gestation placenta and embryo using a 15,000 mouse developmental cDNA microarray. *Proc Natl Acad Sci U S A* **97:** (16) 9127.

Wheeler GN, Hamilton FS, Hoppler S*. 1999. *Univ Dundee, Wellcome Trust Bioctr, Div Cell & Dev Biol, Dundee DD1 5EH, Scotland. Inducible gene expression in transgenic *Xenopus* embryos. *Curr Biol* **10:** (14) 849.

### 15 Technological advances

Bavan S, Ford M*, Kalatzi M. 2000. *Univ Portsmouth, Inst Biomed & Biomol Sci, Ctr Mol Design, King Henry Bldg, King Henry I St, Portsmouth PO1 2DY, England. Genomic and proteomic sequence recognition using a connectionist interference model. *J Chem Technol Biotechnol* **75:** (10) 901.

Botezatu I, Serdyuk O, Potapova G, Shelepov V, Alechina R, Molyaka Y, Ananev V, Bazin I, Garin A, Narimanov M, Knysh V, Melkonyan H, Umansky S*, Lichtenstein A. 2000. *Diagen Corp, 6034 Monterey Ave, Richmond, Ca, USA. Genetic analysis of DNA excreted in urine: A new approach for detecting specific genomic DNA sequences from cells dying in an organism. *Clin Chem* **46:** (8 Pt 1) 1078.

Brady D, Kocic M, Miller AW, Karger BL. 2000. Northeastern Univ, Dept Comp Engn, Boston, Ma 02115, USA. A maximum-likelihood base caller for DNA sequencing. *IEEE Trans Biomed Eng* **47:** (9) 1271.

Bras M, Cloarec JP, Bessueille F, Souteyrand E, Martin JR, Chauvet JP*. 2000. *Ecole Cent Lyon, Equipe Bioingn & Reconnaissance Genet, UMR5621, IFOS PCI, 36 Ave Guy Collongue, FR-69131 Ecully, France. Control of immobilization and hybridization on DNA chips by fluorescence spectroscopy. *J Fluoresc* **10:** (3) 247.

Conrads TP, Anderson GA, Veenstra TD, Pasa-Tolic L, Smith RD*. 2000. *Pacific NW Natl Labs, Environm & Mol Sci Lab, Richland, Wa 99352, USA. Utility of accurate mass tags for proteome-wide protein identification. *Anal Chem* **72:** (14) 3349.

Gillmor SD, Thiel AJ, Strother TC, Smith LM, Lagally MG*. 2000. *Univ Wisconsin, Dept Mat Sci & Engn, 1509 Univ Ave, Madison, Wi 53706, USA. Hydrophilic/hydrophobic patterned surfaces as templates for DNA arrays. *Langmuir* **16:** (18) 7223.

Gottschlich N, Culbertson CT, McKnight TE, Jacobson SC, Ramsey JM*. 2000. *Oak Ridge Natl Lab, POB 2008, Oak Ridge, Tn 37831, USA. Integrated microchip-device for the digestion, separation and postcolumn labeling of proteins and peptides. *J Chromatogr B* **745:** (1) 243.

Hanning A, Westberg J, Roeraade J*. 2000. *Royal Inst Technol, Dept Analyt Chem, SE-10044 Stockholm, Sweden. A liquid core waveguide fluorescence detector for multicapillary electrophoresis applied to DNA sequencing in a 91-capillary array. *Electrophoresis* **21:** (15) 3290.

Hegde P, Qi R, Abernathy K, Gay C, Dharap S, Gaspard R, Hughes JE, Snesrud E, Lee N, Quackenbush J*. 2000. *Inst Genomic Res, 9712 Med Ctr Dr, Rockville, Md 20850, USA. A concise guide to cDNA microarray analysis. *Biotechniques* **29:** (3) 548.

Huang CZ, Li YF, Huang XH, Fan MK. 2000. SW Normal Univ, Inst Environm Chem, CN-400715 Chongqing, Peoples Rep China. Microarray of DNA probes on carboxylate functional beads surface. *Sci China B* **43:** (4) 435.

Ishii M, Hashimoto S, Tsutsumi S, Wada Y, Matsushima K, Kodama T, Aburatani H*. 2000. *Univ Tokyo, Res Ctr Adv Sci & Technol, Meguro ku, 4-6-1 Komaba, Tokyo 153 8904, Japan. Direct comparison of GeneChip and SAGE on the quantitative accuracy in transcript profiling analysis. *Genomics* **68:** (2) 136.

Kumar NV, Bernstein LR*. 2000. *Texas A&M Syst Hlth Sci Ctr, Dept Pathol & Lab Med, 208 Reynolds Med Bldg, College Station, Tx 77843, USA. Screening of a cDNA protein expression library by enhanced chemiluminescence detection. *Biotechniques* **29:** (3) 418.

Leclerc GM, Boockfor FR, Faught WJ, Frawley LS*. 2000. *Med Univ, Dept Cell Biol, Lab Mol Dynam, 171 Ashley Ave, Charleston, SC 29425, USA. Development of a destabilized firefly luciferase enzyme for measurement of gene expression. *Biotechniques* **29:** (3) 590.

Li LJ, Romanova EV, Rubakhin SS, Alexeeva V, Weiss KR, Vilim FS, Sweedler JV*. 2000. *Univ Illinois, Dept Chem, 1209 West Calif St, Urbana, Il 61801, USA. Peptide profiling of cells with multiple gene products: Combining immunochemistry and MALDI mass spectrometry with on-plate microextraction. *Anal Chem* **72:** (16) 3867.

Lichtenwalter KG, Apffel A, Bai J, Chakel JA, Dai YQ, Hahnenberger KM, Li L, Hancock WS*. 2000. *Hewlett Packard Labs, Biomeasurements Grp, Palo Alto, Ca 94304, USA. Approaches to functional genomics: Potential of matrix-assisted laser desorption/ionization-time-of-flight mass spectrometry combined with separation methods for the analysis of DNA in biological samples. *J Chromatogr B* **745:** (1) 231.

MacBeath G, Schreiber SL. 2000. Harvard Univ, Ctr Genome Res, 16 Divin Ave, Cambridge, Ma 02138, USA. Printing proteins as microarrays for high-throughput function determination. *Science* **289:** (5485) 1760.

Natsume T, Nakayama H, Jansson O, Isobe T, Takio K, Mikoshiba K. 2000. Tokyo Metropolitan Univ, Grad Sch Sci, Dept Chem, Integrated Prot Syst Project, Minamiohsawa 1-1, Hachioji, Tokyo 192 0397, Japan. Combination of biomolecular interaction analysis and mass spectrometric amino acid sequencing. *Anal Chem* **72:** (17) 4193.

Nishigaki K, Akasaka K, Hasegawa T. 2000. Saitama Univ, Dept Funct Mat Sci, Urawa, Saitama 338 8570, Japan. Random PCR-based genome sequencing: A non-divide-and-conquer strategy. *DNA Res* **7:** (1) 19.

Ohyama H, Zhang X, Kohno Y, Alevizos I, Posner M, Wong DT, Todd R. 2000. MGH, Dept Oral & Maxillofacial Surg, 1 Fruit St, Boston, Ma 02114, USA. Laser capture microdissection-generated target sample for high-density oligonucleotide array hybridization. *Biotechniques* **29:** (3) 530.

Ponce JR, Perez-Perez JM, Piqueras P, Micol JL*. 2000. *Univ Miguel Hernandez, Div Genet, Campus San Juan, ES-03550 Alicante, Spain. A multiplex reverse transcriptase-polymerase chain reaction method for fluorescence-based semiautomated detection of gene expression in *Arabidopsis thaliana*. *Planta* **211:** (4) 606.

Serebriiskii IG, Toby GG, Golemis EA. 2000. Fox Chase Canc Ctr, 7701 Burholme Ave, Philadelphia, Pa 1911, USA. Streamlined yeast colorimetric reporter activity assays using scanners and plate readers.

Copyright © 2001 John Wiley & Sons, Ltd. 

*Biotechniques* **29:** (2) 278.

Souteyrand E, Cloarec JP, Martin JR, Cabrera M, Bras M, Chauvet JP, Dugas V, Bessueille F. 2000. Ecole Cent Lyon, UMR 5621 CNRS, Lab Ingn & Fonctionalisat Surfaces, 36 Ave Collogue, FR-69130 Ecully, France. Use of microtechnology for DNA chips implementation. *Appl Surf Sci* **164:** 246.

Stears RL, Getts RC, Gullans SR*. 2000. *Brigham & Women's Hosp, Harvard Inst Med, Dept Med, 77 Ave Louis Pasteur, Boston, Ma 02115, USA. A novel, sensitive detection system for high-density microarrays using dendrimer technology. *Physiol Genomics* **3:** (2) 93.

Taton TA, Mirkin CA*, Letsinger RL. 2000. *Northwestern Univ, Dept Chem, Evanston, Il 60208, USA. Scanometric DNA array detection with nanoparticle probes. *Science* **289:** (5485) 1757.

Tian HJ, Brody LC, Landers JP*. 2000. *Univ Virginia, Dept Chem, McCormick Rd, Charlottesville, Va 22901, USA. Rapid detection of deletion, insertion, and substitution mutations *via* heteroduplex analysis using capillary- and microchip-based electrophoresis. *Genome Res* **10:** (9) 1403.

Zhou XC, O'Shea SJ, Li SFY. 2000. Natl Univ Singapore, Inst Mat Res & Engn, SG-119260 Singapore, Singapore. Amplified microgravimetric gene sensor using Au nanoparticle modified oligonucleotides. *Chem Commun* (11) 953.

Zuo X, Speicher DW*. 2000. *Wistar Inst Anat & Biol, 3601 Spruce St, Philadelphia, Pa 19104, USA. A method for global analysis of complex proteomes using sample prefractionation by solution isoelectrofocusing prior to two-dimensional electrophoresis. *Anal Biochem* **284:** (2) 266.

### 16 Bioinformatics

Akmaev VR, Kelley ST, Stormo GD. 2000. Univ Colorado, Dept Appl Math, Box 526, Boulder, Co 80309, USA. Phylogenetically enhanced statistical tools for RNA structure prediction. *Bioinformatics* **16:** (6) 501.

Alter O, Brown PO, Botstein D. 2000. Stanford Univ, Dept Genet, Stanford, Ca 94305, USA. Singular value of decomposition for genome-wide expression data processing and modeling. *Proc Natl Acad Sci U S A* **97:** (18) 10101.

Alves R, Savageau MA*. 2000. *Univ Michigan, Sch Med, Dept Microbiol & Immunol, Med Sci Bldg, Ann Arbor, Mi 48109, USA. Systemic properties of ensembles of metabolic networks: Application of graphical and statistical methods to simple unbranched pathways. *Bioinformatics* **16:** (6) 534.

Bader GD, Hogue CWV*. 2000. *Univ Toronto, Samuel Lunenfeld Res Inst, Dept Biochem, Toronto, Ontario, Canada M5G 1X5. BIND: A data specification for storing and describing biomolecular interactions, molecular complexes and pathways. *Bioinformatics* **16:** (5) 465.

Brett D, Lehmann G, Hanke J, Gross S, Reich J, Bork P. 2000. Max-Delbruck-Ctr Mol Med, Robert Rossle Str 10, DE-13125 Berlin, Germany. EST analysis online: WWW tools for detection of SNPs and alternative splice forms. *Trends Genet* **16:** (9) 416.

Burke DF, Deane CM, Blundell TL. 2000. Univ Cambridge, Dept Biochem, Tennis Court Rd, Cambridge CB1 1QW, England. Browsing the SLoop database of structurally classified loops connecting elements of protein secondary structure. *Bioinformatics* **16:** (6) 513.

Bussemaker HJ, Li H, Siggia ED. 2000. Univ Amsterdam, Swammerdam Inst Life Sci, Kruislaan 318, NL-1098 SM Amsterdam, The Netherlands. Building a dictionary for genomes: Identification of presumptive regulatory sites by statistical analysis. *Proc Natl Acad Sci U S A* **97:** (18) 10096.

Chen T, Skiena SS. 2000. Harvard Univ, Sch Med, Dept Genet, Boston, Ma 02115, USA. A case study in genome-level fragment assembly. *Bioinformatics* **16:** (6) 494.

Drawid A, Gerstein M*. 2000. *Yale Univ, Dept Mol Biophys, 266 Whitney Ave, POB 208114, New Haven, Ct 06520, USA. A Bayesian system integrating expression data with sequence patterns for localizing proteins: Comprehensive application to the yeast genome. *J Mol Biol* **301:** (4) 1059.

Enright AJ, Ouzounis CA*. 2000. *EMBL, European Bioinformat Inst, Res Programme, Computat Gen Grp, Cambridge Outstation, Cambridge CB10 1SD, England. GeneRAGE: A robust algorithm for sequence clustering and domain detection. *Bioinformatics* **16:** (5) 451.

Florea L, Riemer C, Schwartz S, Zhang Z, Stojanovic N, Miller W*, McClelland M. 2000. *Penn State Univ, Dept Comp Sci & Engn, University Park, Pa 16802, USA. Web-based visualization tools for bacterial genome alignments. *Nucleic Acids Res* **28:** (18) 3486.

Frank AC, Lobry JR*. 2000. *Univ Lyon 1, Lab BBE, CNRS UMR 5558, 43 Blvd 11 Novembre 1918, FR-69622 Villeurbanne, France. Oriloc: Prediction of replication boundaries in unannotated bacterial chromosomes. *Bioinformatics* **16:** (6) 560.

Hirosawa M, Ishikawa K, Nagase T, Ohara O*. 2000. *Kazusa DNA Res Inst, Chiba 292 0812, Japan. Detection of spurious interruptions of protein-coding regions in cloned cDNA sequences by GeneMark analysis. *Genome Res* **10:** (9) 1333.

Hooper PM, Zhang HY, Wishart DS. 2000. Univ Alberta, Dept Math Sci, Edmonton, Alberta, Canada T6G 2G1. Prediction of genetic structure in eukaryotic DNA using reference point logistic regression and sequence alignment. *Bioinformatics* **16:** (5) 425.

Jareborg N, Durbin R. 2000. Sanger Ctr, Wellcome Trust Genome Campus, Cambridge CB10 1SA, England. Alfresco: A workbench for comparative genomic sequence analysis. *Genome Res* **10:** (8) 1148.

Jurka J. 2000. Genetic Informat Res Inst, 1170 Morse Ave, Sunnyvale, Ca 94089, USA. Repbase Update: A database and an electronic journal of repetitive elements. *Trends Genet* **16:** (9) 418.

Martin D, Rybicki E. 2000. Univ Cape Town, Dept Microbiol, ZA-7925 Cape Town, Rep Sth Africa. RDP: Detection of recombination amongst aligned sequences. *Bioinformatics* **16:** (6) 562.

McArthur AG, Morrison HG, Nixon JEJ, Passamaneck NQE, Kim U, Hinkle G, Crocker MK, Holder ME, Farr R, Reich CI, Olsen GE, Aley SB, Adam RD, Gillin FS, Sogin ML*. 2000. *Marine Biol Lab, Ctr Comparat Mol Biol & Evolut, Woods Hole, Ma 02543, USA. The *Giardia* genome project database. *FEMS Microbiol Lett* **189:** (2) 271.

Milan D, Hawken R, Cabau C, Leroux S, Genet C, Lahbib Y, Tosser G, Robic A, Hatey F, Alexander L, Beattie C, Schook L, Yerle M, Gellin J. 2000. INRA, Lab Genet Cellulaire, BP 27, FR-31326 Castanet Tolosan, France. IMpRH Server: An RH mapping server available on the Web. *Bioinformatics* **16:** (6) 558.

Park J, Holm L, Heger A, Chothia C. 2000. European Bioinformat Inst, EMBL Outstation, Cambridge CB10 1SD, England. RSDB: Representative protein sequence databases have high information content. *Bioinformatics* **16:** (5) 458.

Paton NW, Khan SA, Hayes A, Moussouni F, Brass A, Eilbeck K, Goble CA, Hubbard SJ, Oliver SG. 2000. Univ Manchester, Dept Comp Sci, Oxford Rd, Manchester M13 9PL, England. Conceptual modelling of genomic information. *Bioinformatics* **16:** (6) 548.

Pesole G, Liuni S, D'Souza M. 2000. Univ Milan, Dipt Fisiol & Biochim Gen, via Celoria 26, IT-20133 Milan, Italy. PatSearch: A pattern matcher software that finds functional elements in nucleotide and protein sequences and assesses their statistical significance. *Bioinformatics* **16:** (5) 439.

Pineda FJ, Lin JS, Fenselau C, Demirev PA. 2000. Johns Hopkins Univ, Appl Phys Lab, Johns Hopkins Rd, Laurel, Md 20723, USA. Testing the significance of microorganism identification by mass spectrometry and proteome database search. *Anal Chem* **72:** (16) 3739.

Pruitt KD, Katz KS, Sicotte H, Maglott DR. 2000. Natl Lib Med, Natl Ctr Biotechnol Informat, Bldg 38A, Room 8N805, Bethesda, Md 20894, USA. Introducing RefSeq and LocusLink: Curated human genome resources at the NCBI. *Trends Genet* **16:** (1) 44.

Snel B, Lehmann G, Bork P, Huynen MA. 2000. European Mol Biol Lab, Meyerhofstr 1, DE-69117 Heidelberg, Germany. STRING: A web-server to retrieve and display the repeatedly occurring neighbourhood of a gene. *Nucleic Acids Res* **28:** (18) 3442.

Zhang CT, Wang J. 2000. Tianjin Univ, Dept Phys, CN-300072 Tianjin, Peoples Rep China. Recognition of protein coding genes in the yeast genome at better than 95% accuracy based on the Z curve. *Nucleic Acids Res* **28:** (14) 2804.

Copyright © 2001 John Wiley & Sons, Ltd. 

## References

[PDF_00952] Akmaev V. R., Kelley S. T., Stormo G. D. (2000). Phylogenetically enhanced statistical tools for RNA structure prediction.. Bioinformatics.

[PDF_00182] Albelda S. M., Sheppard D. (2000). Functional genomics and expression profiling: be there or be square.. Am J Respir Cell Mol Biol.

[PDF_00956] Alter O., Brown P. O., Botstein D. (2000). Singular value decomposition for genome-wide expression data processing and modeling.. Proc Natl Acad Sci U S A.

[PDF_00197] Altshuler D., Pollara V. J., Cowles C. R., Van Etten W. J., Baldwin J., Linton L., Lander E. S. (2000). An SNP map of the human genome generated by reduced representation shotgun sequencing.. Nature.

[PDF_00960] Alves R., Savageau M. A. (2000). Systemic properties of ensembles of metabolic networks: application of graphical and statistical methods to simple unbranched pathways.. Bioinformatics.

[PDF_00589] Anderson K. M., Alrefai W. A., Bonomi P. A., Anderson C. A., Dudeja P., Harris J. E. (2000). A genomic response of H-358 bronchiolar carcinoma cells to MK 886, an inhibitor of 5-lipoxygenase, assessed with a cDNA array.. Anticancer Res.

[PDF_00014] Anderson N. L., Matheson A. D., Steiner S. (2000). Proteomics: applications in basic and applied biology.. Curr Opin Biotechnol.

[PDF_00202] Anderson S. I., Lopez-Corrales N. L., Gorick B., Archibald A. L. (2000). A large-fragment porcine genomic library resource in a BAC vector.. Mamm Genome.

[PDF_00467] Antelmann H., Scharf C., Hecker M. (2000). Phosphate starvation-inducible proteins of Bacillus subtilis: proteomics and transcriptional analysis.. J Bacteriol.

[PDF_00594] Arfin S. M., Long A. D., Ito E. T., Tolleri L., Riehle M. M., Paegle E. S., Hatfield G. W. (2000). Global gene expression profiling in Escherichia coli K12. The effects of integration host factor.. J Biol Chem.

[PDF_00205] Asamizu E., Nakamura Y., Sato S., Tabata S. (2000). A large scale analysis of cDNA in Arabidopsis thaliana: generation of 12,028 non-redundant expressed sequence tags from normalized and size-selected cDNA libraries.. DNA Res.

[PDF_00965] Bader G. D., Hogue C. W. (2000). BIND--a data specification for storing and describing biomolecular interactions, molecular complexes and pathways.. Bioinformatics.

[PDF_00726] Balasubramanian S., Schneider T., Gerstein M., Regan L. (2000). Proteomics of Mycoplasma genitalium: identification and characterization of unannotated and atypical proteins in a small model genome.. Nucleic Acids Res.

[PDF_00018] Baldi P., Brunak S., Chauvin Y., Andersen C. A., Nielsen H. (2000). Assessing the accuracy of prediction algorithms for classification: an overview.. Bioinformatics.

[PDF_00392] Band M. R., Larson J. H., Rebeiz M., Green C. A., Heyen D. W., Donovan J., Windish R., Steining C., Mahyuddin P., Womack J. E. (2000). An ordered comparative map of the cattle and human genomes.. Genome Res.

[PDF_00397] Barbazuk W. B., Korf I., Kadavi C., Heyen J., Tate S., Wun E., Bedell J. A., McPherson J. D., Johnson S. L. (2000). The syntenic relationship of the zebrafish and human genomes.. Genome Res.

[PDF_00026] Barlow K. L., Green J., Clewley J. P. (2000). Viral genome characterisation by the heteroduplex mobility and heteroduplex tracking assays.. Rev Med Virol.

[PDF_00731] Barrett J., Jefferies J. R., Brophy P. M. (2000). Parasite proteomics.. Parasitol Today.

[PDF_00210] Bawden A. L., Glassberg K. J., Diggans J., Shaw R., Farmerie W., Moyer R. W. (2000). Complete genomic sequence of the Amsacta moorei entomopoxvirus: analysis and comparison with other poxviruses.. Virology.

[PDF_00188] Ben-Asher E., Chalifa-Caspi V., Horn-Saban S., Avidan N., Olender Z., Adato A., Glusman G., Safran M., Rubinstein M., Lancet D. (2000). Harvesting the human genome: the Israeli perspective.. Isr Med Assoc J.

[PDF_00401] Bennetzen J. L. (2000). Comparative sequence analysis of plant nuclear genomes:m microcolinearity and its many exceptions.. Plant Cell.

[PDF_00598] Bittner M., Meltzer P., Chen Y., Jiang Y., Seftor E., Hendrix M., Radmacher M., Simon R., Yakhini Z., Ben-Dor A. (2000). Molecular classification of cutaneous malignant melanoma by gene expression profiling.. Nature.

[PDF_00215] Blanc G., Barakat A., Guyot R., Cooke R., Delseny M. (2000). Extensive duplication and reshuffling in the Arabidopsis genome.. Plant Cell.

[PDF_00219] Blank W. A., Stemke G. W. (2000). A physical and genetic map of the Mycoplasma hyopneumoniae strain J genome.. Can J Microbiol.

[PDF_00831] Botezatu I., Serdyuk O., Potapova G., Shelepov V., Alechina R., Molyaka Y., Ananév V., Bazin I., Garin A., Narimanov M. (2000). Genetic analysis of DNA excreted in urine: a new approach for detecting specific genomic DNA sequences from cells dying in an organism.. Clin Chem.

[PDF_00837] Brady D., Kocic M., Miller A. W., Karger B. L. (2000). A maximum-likelihood base caller for DNA sequencing.. IEEE Trans Biomed Eng.

[PDF_00734] Brancia F. L., Oliver S. G., Gaskell S. J. (2000). Improved matrix-assisted laser desorption/ionization mass spectrometric analysis of tryptic hydrolysates of proteins following guanidination of lysine-containing peptides.. Rapid Commun Mass Spectrom.

[PDF_00969] Brett D., Lehmann G., Hanke J., Gross S., Reich J., Bork P. (2000). EST analysis online: WWW tools for detection of SNPs and alternative splice forms.. Trends Genet.

[PDF_00603] Brocklehurst K. R., Morby A. P. (2000). Metal-ion tolerance in Escherichia coli: analysis of transcriptional profiles by gene-array technology.. Microbiology.

[PDF_00973] Burke D. F., Deane C. M., Blundell T. L. (2000). Browsing the SLoop database of structurally classified loops connecting elements of protein secondary structure.. Bioinformatics.

[PDF_00977] Bussemaker H. J., Li H., Siggia E. D. (2000). Building a dictionary for genomes: identification of presumptive regulatory sites by statistical analysis.. Proc Natl Acad Sci U S A.

[PDF_00500] Cacabelos R., Alvarez A., Lombardi V., Fernández-Novoa L., Corzo L., Pérez P., Laredo M., Pichel V., Hernández A., Varela M. (2000). Pharmacological treatment of Alzheimer disease: from psychotropic drugs and cholinesterase inhibitors to pharmacogenomics.. Drugs Today (Barc).

[PDF_00223] Cameron R. A., Mahairas G., Rast J. P., Martinez P., Biondi T. R., Swartzell S., Wallace J. C., Poustka A. J., Livingston B. T., Wray G. A. (2000). A sea urchin genome project: sequence scan, virtual map, and additional resources.. Proc Natl Acad Sci U S A.

[PDF_00982] Chen T., Skiena S. S. (2000). A case study in genome-level fragment assembly.. Bioinformatics.

[PDF_00050] Cho R. J., Campbell M. J. (2000). Transcription, genomes, function.. Trends Genet.

[PDF_00229] Clifford R., Edmonson M., Hu Y., Nguyen C., Scherpbier T., Buetow K. H. (2000). Expression-based genetic/physical maps of single-nucleotide polymorphisms identified by the cancer genome anatomy project.. Genome Res.

[PDF_00846] Conrads T. P., Anderson G. A., Veenstra T. D., Pasa-Tolić L., Smith R. D. (2000). Utility of accurate mass tags for proteome-wide protein identification.. Anal Chem.

[PDF_00234] Dandekar T., Huynen M., Regula J. T., Ueberle B., Zimmermann C. U., Andrade M. A., Doerks T., Sánchez-Pulido L., Snel B., Suyama M. (2000). Re-annotating the Mycoplasma pneumoniae genome sequence: adding value, function and reading frames.. Nucleic Acids Res.

[PDF_00555] Delneri D., Tomlin G. C., Wixon J. L., Hutter A., Sefton M., Louis E. J., Oliver S. G. (2000). Exploring redundancy in the yeast genome: an improved strategy for use of the cre-loxP system.. Gene.

[PDF_00053] Destenaves B., Thomas F. (2000). New advances in pharmacogenomics.. Curr Opin Chem Biol.

[PDF_00985] Drawid A., Gerstein M. (2000). A Bayesian system integrating expression data with sequence patterns for localizing proteins: comprehensive application to the yeast genome.. J Mol Biol.

[PDF_00607] Eickhoff H., Schuchhardt J., Ivanov I., Meier-Ewert S., O'Brien J., Malik A., Tandon N., Wolski E. W., Rohlfs E., Nyarsik L. (2000). Tissue gene expression analysis using arrayed normalized cDNA libraries.. Genome Res.

[PDF_00612] Endo M., Kokubun T., Takahata Y., Higashitani A., Tabata S., Watanabe M. (2000). Analysis of expressed sequence tags of flower buds in Lotus japonicus.. DNA Res.

[PDF_00244] Endrizzi M. G., Hadinoto V., Growney J. D., Miller W., Dietrich W. F. (2000). Genomic sequence analysis of the mouse Naip gene array.. Genome Res.

[PDF_00062] Farkas G., Leibovitch B. A., Elgin S. C. (2000). Chromatin organization and transcriptional control of gene expression in Drosophila.. Gene.

[PDF_00066] Fenyö D. (2000). Identifying the proteome: software tools.. Curr Opin Biotechnol.

[PDF_00616] Fidock D. A., Nguyen T. V., Ribeiro J. M., Valenzuela J. G., James A. A. (2000). Plasmodium falciparum: generation of a cDNA library enriched in sporozoite-specific transcripts by directional tag subtractive hybridization.. Exp Parasitol.

[PDF_00994] Florea L., Riemer C., Schwartz S., Zhang Z., Stojanovic N., Miller W., McClelland M. (2000). Web-based visualization tools for bacterial genome alignments.. Nucleic Acids Res.

[PDF_00405] Forozan F., Mahlamäki E. H., Monni O., Chen Y., Veldman R., Jiang Y., Gooden G. C., Ethier S. P., Kallioniemi A., Kallioniemi O. P. (2000). Comparative genomic hybridization analysis of 38 breast cancer cell lines: a basis for interpreting complementary DNA microarray data.. Cancer Res.

[PDF_00255] Frohme M., Camargo A. A., Heber S., Czink C., Simpson A. J., Hoheisel J. D., de Souza A. P. (2000). Mapping analysis of the Xylella fastidiosa genome.. Nucleic Acids Res.

[PDF_00622] Gabrielsson B. L., Carlsson B., Carlsson L. M. (2000). Partial genome scale analysis of gene expression in human adipose tissue using DNA array.. Obes Res.

[PDF_00535] Gehring A. M., Nodwell J. R., Beverley S. M., Losick R. (2000). Genomewide insertional mutagenesis in Streptomyces coelicolor reveals additional genes involved in morphological differentiation.. Proc Natl Acad Sci U S A.

[PDF_00854] Gottschlich N., Culbertson C. T., McKnight T. E., Jacobson S. C., Ramsey J. M. (2000). Integrated microchip-device for the digestion, separation and postcolumn labeling of proteins and peptides.. J Chromatogr B Biomed Sci Appl.

[PDF_00411] Goureau A., Vignoles M., Pinton P., Gellin J., Yerle M. (2000). Improvement of comparative map between porcine chromosomes 1 and 7 and human chromosomes 6, 14, and 15 by using human YACs.. Mamm Genome.

[PDF_00416] Grigoriev A. (2000). Graphical genome comparison: rearrangements and replication origin of Helicobacter pylori.. Trends Genet.

[PDF_00797] Guo D., Wu Y., Kaplan H. B. (2000). Identification and characterization of genes required for early Myxococcus xanthus developmental gene expression.. J Bacteriol.

[PDF_00740] Gygi S. P., Corthals G. L., Zhang Y., Rochon Y., Aebersold R. (2000). Evaluation of two-dimensional gel electrophoresis-based proteome analysis technology.. Proc Natl Acad Sci U S A.

[PDF_00081] Gygi S. P., Rist B., Aebersold R. (2000). Measuring gene expression by quantitative proteome analysis.. Curr Opin Biotechnol.

[PDF_00859] Hanning A., Westberg J., Roeraade J. (2000). A liquid core waveguide fluorescence detector for multicapillary electrophoresis applied to DNA sequencing in a 91-capillary array.. Electrophoresis.

[PDF_00333] Hansmann S., Martin W. (2000). Phylogeny of 33 ribosomal and six other proteins encoded in an ancient gene cluster that is conserved across prokaryotic genomes: influence of excluding poorly alignable sites from analysis.. Int J Syst Evol Microbiol.

[PDF_00864] Hegde P., Qi R., Abernathy K., Gay C., Dharap S., Gaspard R., Hughes J. E., Snesrud E., Lee N., Quackenbush J. (2000). A concise guide to cDNA microarray analysis.. Biotechniques.

[PDF_00260] Heidelberg J. F., Eisen J. A., Nelson W. C., Clayton R. A., Gwinn M. L., Dodson R. J., Haft D. H., Hickey E. K., Peterson J. D., Umayam L. (2000). DNA sequence of both chromosomes of the cholera pathogen Vibrio cholerae.. Nature.

[PDF_01002] Hirosawa M., Ishikawa K., Nagase T., Ohara O. (2000). Detection of spurious interruptions of protein-coding regions in cloned cDNA sequences by GeneMark analysis.. Genome Res.

[PDF_00632] Hong T. M., Yang P. C., Peck K., Chen J. J., Yang S. C., Chen Y. C., Wu C. W. (2000). Profiling the downstream genes of tumor suppressor PTEN in lung cancer cells by complementary DNA microarray.. Am J Respir Cell Mol Biol.

[PDF_01006] Hooper P. M., Zhang H., Wishart D. S. (2000). Prediction of genetic structure in eukaryotic DNA using reference point logistic regression and sequence alignment.. Bioinformatics.

[PDF_00530] Hrabé de Angelis M. H., Flaswinkel H., Fuchs H., Rathkolb B., Soewarto D., Marschall S., Heffner S., Pargent W., Wuensch K., Jung M. (2000). Genome-wide, large-scale production of mutant mice by ENU mutagenesis.. Nat Genet.

[PDF_00338] Hurst L. D., Randerson J. P. (2000). Dosage, deletions and dominance: simple models of the evolution of gene expression.. J Theor Biol.

[PDF_00085] Huynen M., Snel B., Lathe W., Bork P. (2000). Predicting protein function by genomic context: quantitative evaluation and qualitative inferences.. Genome Res.

[PDF_00637] Ichikawa J. K., Norris A., Bangera M. G., Geiss G. K., van 't Wout A. B., Bumgarner R. E., Lory S. (2000). Interaction of pseudomonas aeruginosa with epithelial cells: identification of differentially regulated genes by expression microarray analysis of human cDNAs.. Proc Natl Acad Sci U S A.

[PDF_00872] Ishii M., Hashimoto S., Tsutsumi S., Wada Y., Matsushima K., Kodama T., Aburatani H. (2000). Direct comparison of GeneChip and SAGE on the quantitative accuracy in transcript profiling analysis.. Genomics.

[PDF_00265] Janssens W., Salminen M. O., Laukkanen T., Heyndrickx L., van der Auwera, Colebunders R., McCutchan F. E., van der Groen G. (2000). Near full-length genome analysis of HIV type 1 CRF02.AG subtype C and CRF02.AG subtype G recombinants.. AIDS Res Hum Retroviruses.

[PDF_01010] Jareborg N., Durbin R. (2000). Alfresco--a workbench for comparative genomic sequence analysis.. Genome Res.

[PDF_00744] Ji J., Chakraborty A., Geng M., Zhang X., Amini A., Bina M., Regnier F. (2000). Strategy for qualitative and quantitative analysis in proteomics based on signature peptides.. J Chromatogr B Biomed Sci Appl.

[PDF_01013] Jurka J. (2000). Repbase update: a database and an electronic journal of repetitive elements.. Trends Genet.

[PDF_00471] Kaebernick M., Neilan B. A., Börner T., Dittmann E. (2000). Light and the transcriptional response of the microcystin biosynthesis gene cluster.. Appl Environ Microbiol.

[PDF_00801] Kelsh R. N., Schmid B., Eisen J. S. (2000). Genetic analysis of melanophore development in zebrafish embryos.. Dev Biol.

[PDF_00540] Kennerdell J. R., Carthew R. W. (2000). Heritable gene silencing in Drosophila using double-stranded RNA.. Nat Biotechnol.

[PDF_00419] Kent W. J., Zahler A. M. (2000). Conservation, regulation, synteny, and introns in a large-scale C. briggsae-C. elegans genomic alignment.. Genome Res.

[PDF_00564] Kong H., Lin L. F., Porter N., Stickel S., Byrd D., Posfai J., Roberts R. J. (2000). Functional analysis of putative restriction-modification system genes in the Helicobacter pylori J99 genome.. Nucleic Acids Res.

[PDF_00748] Kristensen D. B., Kawada N., Imamura K., Miyamoto Y., Tateno C., Seki S., Kuroki T., Yoshizato K. (2000). Proteome analysis of rat hepatic stellate cells.. Hepatology.

[PDF_00345] Kruglyak S., Durrett R., Schug M. D., Aquadro C. F. (2000). Distribution and abundance of microsatellites in the yeast genome can Be explained by a balance between slippage events and point mutations.. Mol Biol Evol.

[PDF_00423] Ku H. M., Vision T., Liu J., Tanksley S. D. (2000). Comparing sequenced segments of the tomato and Arabidopsis genomes: large-scale duplication followed by selective gene loss creates a network of synteny.. Proc Natl Acad Sci U S A.

[PDF_00475] Kubo M., Kakimoto T. (2000). The Cytokinin-hypersensitive genes of Arabidopsis negatively regulate the cytokinin-signaling pathway for cell division and chloroplast development.. Plant J.

[PDF_00648] Kudoh K., Ramanna M., Ravatn R., Elkahloun A. G., Bittner M. L., Meltzer P. S., Trent J. M., Dalton W. S., Chin K. V. (2000). Monitoring the expression profiles of doxorubicin-induced and doxorubicin-resistant cancer cells by cDNA microarray.. Cancer Res.

[PDF_00881] Leclerc G. M., Boockfor F. R., Faught W. J., Frawley L. S. (2000). Development of a destabilized firefly luciferase enzyme for measurement of gene expression.. Biotechniques.

[PDF_00092] Legrain P., Jestin J. L., Schächter V. (2000). From the analysis of protein complexes to proteome-wide linkage maps.. Curr Opin Biotechnol.

[PDF_00885] Li L., Romanova E. V., Rubakhin S. S., Alexeeva V., Weiss K. R., Vilim F. S., Sweedler J. V. (2000). Peptide profiling of cells with multiple gene products: combining immunochemistry and MALDI mass spectrometry with on-plate microextraction.. Anal Chem.

[PDF_00661] Liang F., Holt I., Pertea G., Karamycheva S., Salzberg S. L., Quackenbush J. (2000). An optimized protocol for analysis of EST sequences.. Nucleic Acids Res.

[PDF_00890] Lichtenwalter K. G., Apffel A., Bai J., Chakel J. A., Dai Y., Hahnenberger K. M., Li L., Hancock W. S. (2000). Approaches to functional genomics: potential of matrix-assisted laser desorption ionization--time of flight mass spectrometry combined with separation methods for the analysis of DNA in biological samples.. J Chromatogr B Biomed Sci Appl.

[PDF_00665] Livesey F. J., Furukawa T., Steffen M. A., Church G. M., Cepko C. L. (2000). Microarray analysis of the transcriptional network controlled by the photoreceptor homeobox gene Crx.. Curr Biol.

[PDF_00355] Lo N., Tokuda G., Watanabe H., Rose H., Slaytor M., Maekawa K., Bandi C., Noda H. (2000). Evidence from multiple gene sequences indicates that termites evolved from wood-feeding cockroaches.. Curr Biol.

[PDF_00511] Loging W. T., Lal A., Siu I. M., Loney T. L., Wikstrand C. J., Marra M. A., Prange C., Bigner D. D., Strausberg R. L., Riggins G. J. (2000). Identifying potential tumor markers and antigens by database mining and rapid expression screening.. Genome Res.

[PDF_00670] Lombardía L. J., Cadahía-Rodríguez J. L., Freire-Picos M. A., González-Siso M. I., Rodríguez-Torres A. M., Cerdán M. E. (2000). Transcript analysis of 203 novel genes from Saccharomyces cerevisiae in hap1 and rox1 mutant backgrounds.. Genome.

[PDF_00896] MacBeath G., Schreiber S. L. (2000). Printing proteins as microarrays for high-throughput function determination.. Science.

[PDF_01016] Martin D., Rybicki E. (2000). RDP: detection of recombination amongst aligned sequences.. Bioinformatics.

[PDF_01019] McArthur A. G., Morrison H. G., Nixon J. E., Passamaneck N. Q., Kim U., Hinkle G., Crocker M. K., Holder M. E., Farr R., Reich C. I. (2000). The Giardia genome project database.. FEMS Microbiol Lett.

[PDF_00276] McCarthy L. C., Bihoreau M. T., Kiguwa S. L., Browne J., Watanabe T. K., Hishigaki H., Tsuji A., Kiel S., Webber C., Davis M. E. (2000). A whole-genome radiation hybrid panel and framework map of the rat genome.. Mamm Genome.

[PDF_00681] McDonagh P. D., Myler P. J., Stuart K. (2000). The unusual gene organization of Leishmania major chromosome 1 may reflect novel transcription processes.. Nucleic Acids Res.

[PDF_00753] McKerrow J. H., Bhargava V., Hansell E., Huling S., Kuwahara T., Matley M., Coussens L., Warren R. (2000). A functional proteomics screen of proteases in colorectal carcinoma.. Mol Med.

[PDF_00516] Mei R., Galipeau P. C., Prass C., Berno A., Ghandour G., Patil N., Wolff R. K., Chee M. S., Reid B. J., Lockhart D. J. (2000). Genome-wide detection of allelic imbalance using human SNPs and high-density DNA arrays.. Genome Res.

[PDF_00102] Meldrum D. (2000). Automation for genomics, part one: preparation for sequencing.. Genome Res.

[PDF_00105] Meldrum D. (2000). Automation for genomics, part two: sequencers, microarrays, and future trends.. Genome Res.

[PDF_00282] Melkerson-Watson L. J., Rode C. K., Zhang L., Foxman B., Bloch C. A. (2000). Integrated genomic map from uropathogenic Escherichia coli J96.. Infect Immun.

[PDF_01024] Milan D., Hawken R., Cabau C., Leroux S., Genet C., Lahbib Y., Tosser G., Robic A., Hatey F., Alexander L. (2000). IMpRH server: an RH mapping server available on the Web.. Bioinformatics.

[PDF_00436] Muniyappa K., Vaze M. B., Ganesh N., Sreedhar Reddy M., Guhan N., Venkatesh R. (2000). Comparative genomics of Mycobacterium tuberculosis and Escherichia coli for recombination (rec) genes.. Microbiology.

[PDF_00121] Myler P. J., Stuart K. D. (2000). Recent developments from the Leishmania genome project.. Curr Opin Microbiol.

[PDF_00479] Nadimpalli R., Yalpani N., Johal G. S., Simmons C. R. (2000). Prohibitins, stomatins, and plant disease response genes compose a protein superfamily that controls cell proliferation, ion channel regulation, and death.. J Biol Chem.

[PDF_00286] Nagase T., Kikuno R., Nakayama M., Hirosawa M., Ohara O. (2000). Prediction of the coding sequences of unidentified human genes. XVIII. The complete sequences of 100 new cDNA clones from brain which code for large proteins in vitro.. DNA Res.

[PDF_00685] Nakachi N., Matsunaga K., Klein T. W., Friedman H., Yamamoto Y. (2000). Differential effects of virulent versus avirulent Legionella pneumophila on chemokine gene expression in murine alveolar macrophages determined by cDNA expression array technique.. Infect Immun.

[PDF_00758] Natera S. H., Guerreiro N., Djordjevic M. A. (2000). Proteome analysis of differentially displayed proteins as a tool for the investigation of symbiosis.. Mol Plant Microbe Interact.

[PDF_00900] Natsume T., Nakayama H., Jansson O., Isobe T., Takio K., Mikoshiba K. (2000). Combination of biomolecular interaction analysis and mass spectrometric amino acid sequencing.. Anal Chem.

[PDF_00691] Nikaido I., Asamizu E., Nakajima M., Nakamura Y., Saga N., Tabata S. (2000). Generation of 10,154 expressed sequence tags from a leafy gametophyte of a marine red alga, Porphyra yezoensis.. DNA Res.

[PDF_00544] Nolan P. M., Peters J., Strivens M., Rogers D., Hagan J., Spurr N., Gray I. C., Vizor L., Brooker D., Whitehill E. (2000). A systematic, genome-wide, phenotype-driven mutagenesis programme for gene function studies in the mouse.. Nat Genet.

[PDF_00440] O'Neill C. M., Bancroft I. (2000). Comparative physical mapping of segments of the genome of Brassica oleracea var. alboglabra that are homoeologous to sequenced regions of chromosomes 4 and 5 of Arabidopsis thaliana.. Plant J.

[PDF_00909] Ohyama H., Zhang X., Kohno Y., Alevizos I., Posner M., Wong D. T., Todd R. (2000). Laser capture microdissection-generated target sample for high-density oligonucleotide array hybridization.. Biotechniques.

[PDF_00569] Pace T., Scotti R., Janse C. J., Waters A. P., Birago C., Ponzi M. (2000). Targeted terminal deletions as a tool for functional genomics studies in Plasmodium.. Genome Res.

[PDF_00124] Paigen K., Eppig J. T. (2000). A mouse phenome project.. Mamm Genome.

[PDF_01029] Park J., Holm L., Heger A., Chothia C. (2000). RSDB: representative protein sequence databases have high information content.. Bioinformatics.

[PDF_01033] Paton N. W., Khan S. A., Hayes A., Moussouni F., Brass A., Eilbeck K., Goble C. A., Hubbard S. J., Oliver S. G. (2000). Conceptual modelling of genomic information.. Bioinformatics.

[PDF_00695] Peek R. M., van Doorn L. J., Donahue J. P., Tham K. T., Figueiredo C., Blaser M. J., Miller G. G. (2000). Quantitative detection of Helicobacter pylori gene expression in vivo and relationship to gastric pathology.. Infect Immun.

[PDF_01037] Pesole G., Liuni S., D'Souza M. (2000). PatSearch: a pattern matcher software that finds functional elements in nucleotide and protein sequences and assesses their statistical significance.. Bioinformatics.

[PDF_00805] Petkov P. M., Kim K., Sandhu J., Shafritz D. A., Dabeva M. D. (2000). Identification of differentially expressed genes in epithelial stem/progenitor cells of fetal rat liver.. Genomics.

[PDF_01042] Pineda F. J., Lin J. S., Fenselau C., Demirev P. A. (2000). Testing the significance of microorganism identification by mass spectrometry and proteome database search.. Anal Chem.

[PDF_00444] Plant M. L., Plant M. A. (1988). Maternal use of alcohol and other drugs during pregnancy and birth abnormalities: further results from a prospective study.. Alcohol Alcohol.

[PDF_00914] Ponce M. R., Pérez-Pérez J. M., Piqueras P., Micol J. L. (2000). A multiplex reverse transcriptase-polymerase chain reaction method for fluorescence-based semiautomated detection of gene expression in Arabidopsis thaliana.. Planta.

[PDF_00810] Popovici R. M., Kao L. C., Giudice L. C. (2000). Discovery of new inducible genes in in vitro decidualized human endometrial stromal cells using microarray technology.. Endocrinology.

[PDF_00573] Porcel B. M., Aslund L., Pettersson U., Andersson B. (2000). Trypanosoma cruzi: a putative vacuolar ATP synthase subunit and a CAAX prenyl protease-encoding gene, as examples of gene identification in genome projects.. Exp Parasitol.

[PDF_00291] Porcel B. M., Tran A. N., Tammi M., Nyarady Z., Rydâker M., Urmenyi T. P., Rondinelli E., Pettersson U., Andersson B., Aslund L. (2000). Gene survey of the pathogenic protozoan Trypanosoma cruzi.. Genome Res.

[PDF_00134] Riechmann J. L., Ratcliffe O. J. (2000). A genomic perspective on plant transcription factors.. Curr Opin Plant Biol.

[PDF_00137] Rockey D. D., Lenart J., Stephens R. S. (2000). Genome sequencing and our understanding of chlamydiae.. Infect Immun.

[PDF_00296] Ruepp A., Graml W., Santos-Martinez M. L., Koretke K. K., Volker C., Mewes H. W., Frishman D., Stocker S., Lupas A. N., Baumeister W. (2000). The genome sequence of the thermoacidophilic scavenger Thermoplasma acidophilum.. Nature.

[PDF_00362] Schmitz J., Ohme M., Zischler H. (2000). The complete mitochondrial genome of Tupaia belangeri and the phylogenetic affiliation of scandentia to other eutherian orders.. Mol Biol Evol.

[PDF_00305] Siegel A. F., van den Engh G., Hood L., Trask B., Roach J. C. (2000). Modeling the feasibility of whole genome shotgun sequencing using a pairwise end strategy.. Genomics.

[PDF_00763] Siegel R. W., Allen B., Pavlik P., Marks J. D., Bradbury A. (2000). Mass spectral analysis of a protein complex using single-chain antibodies selected on a peptide target: applications to functional genomics.. J Mol Biol.

[PDF_01050] Snel B., Lehmann G., Bork P., Huynen M. A. (2000). STRING: a web-server to retrieve and display the repeatedly occurring neighbourhood of a gene.. Nucleic Acids Res.

[PDF_00147] Sniegowski P. (1999). The genomics of adaptation in yeast.. Curr Biol.

[PDF_00149] Springer P. S. (2000). Gene traps: tools for plant development and genomics.. Plant Cell.

[PDF_00701] Steimer A., Amedeo P., Afsar K., Fransz P., Mittelsten Scheid O., Paszkowski J. (2000). Endogenous targets of transcriptional gene silencing in Arabidopsis.. Plant Cell.

[PDF_00314] Steinmetz L. M., Mindrinos M., Oefner P. J. (2000). Combining genome sequences and new technologies for dissecting the genetics of complex phenotypes.. Trends Plant Sci.

[PDF_00318] Stover C. K., Pham X. Q., Erwin A. L., Mizoguchi S. D., Warrener P., Hickey M. J., Brinkman F. S., Hufnagle W. O., Kowalik D. J., Lagrou M. (2000). Complete genome sequence of Pseudomonas aeruginosa PAO1, an opportunistic pathogen.. Nature.

[PDF_00450] Tan S. H., Nishiguchi M., Murata M., Motoyoshi F. (2000). The genome structure of kyuri green mottle mosaic tobamovirus and its comparison with that of cucumber green mottle mosaic tobamovirus.. Arch Virol.

[PDF_00815] Tanaka T. S., Jaradat S. A., Lim M. K., Kargul G. J., Wang X., Grahovac M. J., Pantano S., Sano Y., Piao Y., Nagaraja R. (2000). Genome-wide expression profiling of mid-gestation placenta and embryo using a 15,000 mouse developmental cDNA microarray.. Proc Natl Acad Sci U S A.

[PDF_00324] Tanno F., Nakatsu A., Toriyama S., Kojima M. (2000). Complete nucleotide sequence of Northern cereal mosaic virus and its genome organization.. Arch Virol.

[PDF_00934] Taton T. A., Mirkin C. A., Letsinger R. L. (2000). Scanometric DNA array detection with nanoparticle probes.. Science.

[PDF_00705] Taylor L. A., Carthy C. M., Yang D., Saad K., Wong D., Schreiner G., Stanton L. W., McManus B. M. (2000). Host gene regulation during coxsackievirus B3 infection in mice: assessment by microarrays.. Circ Res.

[PDF_00937] Tian H., Brody L. C., Landers J. P. (2000). Rapid detection of deletion, insertion, and substitution mutations via heteroduplex analysis using capillary- and microchip-based electrophoresis.. Genome Res.

[PDF_00152] Tjalsma H., Bolhuis A., Jongbloed J. D., Bron S., van Dijl J. M. (2000). Signal peptide-dependent protein transport in Bacillus subtilis: a genome-based survey of the secretome.. Microbiol Mol Biol Rev.

[PDF_00455] Voigt B., Kitada K., Klöting I., Serikawa T. (2000). Genetic comparison between laboratory rats and Japanese and German wild rats.. Mamm Genome.

[PDF_00373] Vrati S. (2000). Comparison of the genome sequences and the phylogenetic analyses of the GP78 and the Vellore P20778 isolates of Japanese encephalitis virus from India.. J Biosci.

[PDF_00711] Wang R., Guegler K., LaBrie S. T., Crawford N. M. (2000). Genomic analysis of a nutrient response in Arabidopsis reveals diverse expression patterns and novel metabolic and potential regulatory genes induced by nitrate.. Plant Cell.

[PDF_00821] Wheeler G. N., Hamilton F. S., Hoppler S. (2000). Inducible gene expression in transgenic Xenopus embryos.. Curr Biol.

[PDF_00378] Yang F., Graphodatsky A. S., O'Brien P. C., Colabella A., Solanky N., Squire M., Sargan D. R., Ferguson-Smith M. A. (2000). Reciprocal chromosome painting illuminates the history of genome evolution of the domestic cat, dog and human.. Chromosome Res.

[PDF_00716] Ye R. W., Tao W., Bedzyk L., Young T., Chen M., Li L. (2000). Global gene expression profiles of Bacillus subtilis grown under anaerobic conditions.. J Bacteriol.

[PDF_00720] Yoshikawa T., Nagasugi Y., Azuma T., Kato M., Sugano S., Hashimoto K., Masuho Y., Muramatsu M., Seki N. (2000). Isolation of novel mouse genes differentially expressed in brain using cDNA microarray.. Biochem Biophys Res Commun.

[PDF_00773] Yu L. R., Zeng R., Shao X. X., Wang N., Xu Y. H., Xia Q. C. (2000). Identification of differentially expressed proteins between human hepatoma and normal liver cell lines by two-dimensional electrophoresis and liquid chromatography-ion trap mass spectrometry.. Electrophoresis.

[PDF_00328] Yuan Q., Liang F., Hsiao J., Zismann V., Benito M. I., Quackenbush J., Wing R., Buell R. (2000). Anchoring of rice BAC clones to the rice genetic map in silico.. Nucleic Acids Res.

[PDF_01054] Zhang C. T., Wang J. (2000). Recognition of protein coding genes in the yeast genome at better than 95% accuracy based on the Z curve.. Nucleic Acids Res.

[PDF_00946] Zuo X., Speicher D. W. (2000). A method for global analysis of complex proteomes using sample prefractionation by solution isoelectrofocusing prior to two-dimensional electrophoresis.. Anal Biochem.

